# Generation of mice with longer and better preserved telomeres in the absence of genetic manipulations

**DOI:** 10.1038/ncomms11739

**Published:** 2016-06-02

**Authors:** Elisa Varela, Miguel A. Muñoz-Lorente, Agueda M. Tejera, Sagrario Ortega, Maria A. Blasco

**Affiliations:** 1 Telomeres and Telomerase Group, Molecular Oncology Program, Spanish National Cancer Research Centre (CNIO), Melchor Fernández Almagro 3, Madrid E-28029, Spain; 2 Transgenics Mice Unit, Biotechnology Program, Spanish National Cancer Research Centre (CNIO), Melchor Fernández Almagro 3, Madrid E-28029, Spain

## Abstract

Although telomere length is genetically determined, mouse embryonic stem (ES) cells with telomeres of twice the normal size have been generated. Here, we use such ES cells with ‘hyper-long' telomeres, which also express green fluorescent protein (GFP), to generate chimaeric mice containing cells with both hyper-long and normal telomeres. We show that chimaeric mice contain GFP-positive cells in all mouse tissues, display normal tissue histology and normal survival. Both hyper-long and normal telomeres shorten with age, but GFP-positive cells retain longer telomeres as mice age. Chimaeric mice with hyper-long telomeres also accumulate fewer cells with short telomeres and less DNA damage with age, and express lower levels of p53. In highly renewing compartments, such as the blood, cells with hyper-long telomeres are longitudinally maintained or enriched with age. We further show that wound-healing rates in the skin are increased in chimaeric mice. Our work demonstrates that mice with functional, longer and better preserved telomeres can be generated without the need for genetic manipulations, such as *TERT* overexpression.

## Abstract (web summary)

Telomere shortening has been linked to some aspects of organismal ageing. Here the authors create chimaeric mice that contain a mix of cells with normal or unnaturally long telomeres, and show chimaeric mice are protected from some forms of ageing-associated cellular damage and have accelerated wound-healing.

## Graphical abstract

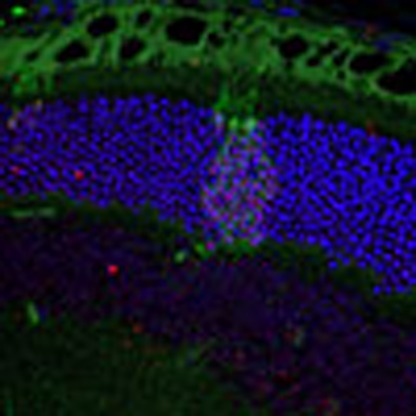

Telomeres are nucleoprotein structures localized at the ends of chromosomes, which in vertebrates consist of tandem repeats of the TTAGGG DNA sequence ending in a 3′overhang of the G-rich strand^1^,^2^. Telomeres protect chromosome ends from degradation and DNA repair activities and are essential for chromosome stability^1^,^3^,^4^. The shelterin complex, composed of six proteins, binds telomeric repeats and is involved in telomere protection and telomere length regulation^1^,^5^. Telomere length decreases associated to cell division due to the inability of the replication machinery to copy the very ends of chromosomes^6^,^7^. Telomerase, a reverse transcriptase, can add de novo repeats onto telomere ends^8^,^9^ and in this manner can elongate or maintain telomeres in cells where it is active, such as cancer cells and pluripotent stem cells^10^,^11^,^12^,^13^. In the adult organism, telomerase activity is restricted to adult stem cell compartments, which also show the longest telomeres; however, this is not sufficient to prevent progressive telomere shortening with ageing^14^,^15^,^16^. When telomeres become critically short, they impair stem cell function^17^ and the regenerative capacity of tissues^12^. At the molecular level, critically short telomeres trigger a persistent DNA damage response that leads to senescence or apoptosis^18^,^19^. There is mounting genetic evidence supporting the notion that accumulation of critically short telomeres leads to ageing and ageing-associated diseases both in mice and humans^20^,^21^,^22^,^23^,^24^,^25^. In particular, telomerase-deficient mice, which show accelerated telomere shortening owing to telomerase deficiency, display organ dysfunction and accelerated ageing^24^,^26^. A similar phenotype is observed in human diseases with mutations in telomerase^20^,^25^,^27^. Telomere length is genetically determined and varies inter species^28^,^29^,^30^,^31^. In addition, the rate of telomere shortening and of accumulation of short telomeres with age also varies between species. In particular, while humans are born with an average telomere length of ∼10–15 kb and they shorten at a rate of ∼70 bp per year, mice of the *Mus musculus C57BL6* mouse strain, are born with telomeres ∼40–50 kb at birth, which shorten 100-times faster than in humans, while the average telomere length in dogs varies between 11.4 and 27.9 kb and telomeres shorten 10-fold faster than in humans^16^,^31^,^32^. Furthermore, the accumulation of short telomeres is predictive of individual longevity both in birds and mice^31^,^33^.

We and others recently described that telomerase is activated and telomeres elongated in the inner cell mass (ICM) of the blastocyst in mice, where it resets the normal telomere length of the unmodified species^34^,^35^. Interestingly, we also found that *in vitro* expansion of mouse embryonic stem (ES) cells derived from the ICM results in a further telomere lengthening until doubling the normal telomere length of the ICM of the blastocyst, a process that is associated to loss of heterochromatic marks^35^. Importantly, this telomere elongation occurs in the absence of modifications in the telomerase gene. These *in vitro* generated telomeres are referred to as hyper-long telomeres^35^. Interestingly, ES cells with hyper-long telomeres can be aggregated in morulae, and can undergo development to the blastocyst stage maintaining the hyper-long telomere phenotype^35^.

Here, we set to address the hypothesis of whether it would be possible to modify the normal telomere length of the *M. musculus* species by altering telomere length in ES cell in the absence of genetic manipulations. Thus, we set to study whether ES cell with hyper-long telomeres owing to *in vitro* passaging, were able to give rise to chimaeric mice composed of cells with hyper-long telomeres, and to study telomere length dynamics with ageing in these mice.

Our results indicate that it is possible to generate mice bearing cells with hyper-long telomeres and that these cells contribute to the normal architecture and function of adult organs. Interestingly, the accumulation of short telomeres with ageing is reduced in cells bearing hyper-long telomeres, which in turn results in lower accumulation of DNA damage in tissues with age. In addition, cells with hyper-long telomeres are maintained with ageing in highly proliferative compartments like blood, as well as are able to heal skin wounds faster than cells bearing normal length telomeres. These findings demonstrate that it is possible to modify the telomere length of a mammalian species in the absence of any genetic manipulation. This opens the possibility of altering the rate of accumulation of short telomeres with ageing in the absence of changes in telomerase expression, thus avoiding its potentially undesired effects in facilitating cancer.

## Results

### Few passages required for ES cells with hyper-long telomeres

**Figure 1:**
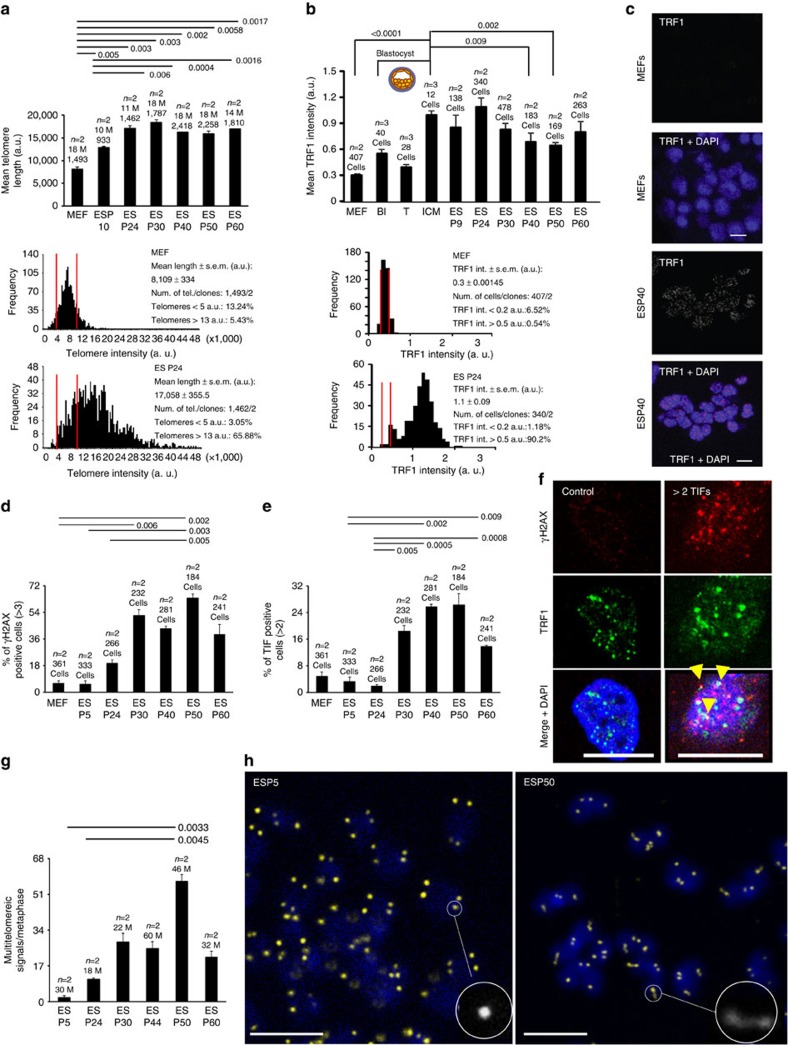
Analysis of ES cells bearing hyper-long telomeres. (**a**) Mean telomere length analysed on metaphase spreads in primary MEF (passage 2) and ES cells at the indicated passages. The ‘M' in the graph indicates the number of metaphases studied. Underneath, the two graphs show the histograms corresponding to telomere signals in the primary MEFs and ES cells at passage 24. (**b**) Mean TRF1 intensity in primary MEFs (passage 2), blastocysts and ES cells at the indicated passages. Underneath, the graphs show the histograms for TRF1 intensity in MEFs and ES cells at passage 24. (**c**) Representative micrographs of TRF1 stain in selected samples described in **b**. Scale bar, 10 μm. (**d**) Per cent of cells with DNA damage measured by IF with anti γH2AX antibody in primary MEFs (passage 2) and ES cells at the indicated passages. We show a representative graph of three independent experiments. (**e**) Per cent of cells with more than two TIFs, analysed by IF with anti γH2AX and TRF1 antibodies in primary MEFs (passage 2) and ES cells at the indicated passages. We show a representative graph of three independent experiments. (**f**) Representative micrographs of control or damaged cells. Cells were subjected to IF with anti γH2AX and TRF1 antibodies. Co-localization of γH2AX and TRF1 is indicated with yellow arrows. Scale bar, 10 μm. (**g**) Mean number of MTS in metaphases from ES cells at the indicated passages, analysed by telomere FISH. Representative graph of two independent experiments. (**h**) Representative images of metaphases at passages 5 and 50. A representative telomere shape is highlighted in each micrograph. Scale bar, 5 μm. *n*=number of independent primary MEFs or clones of ES cells. The s.e.m. was represented in error bars. Student *t*-test with the Bonferroni correction was used to calculate the *P* values. MEF, mouse embryonic fibroblasts.

We showed previously that ES cells lengthen their telomeres upon *in vitro* expansion^35^. Thus, here we first addressed whether there was a limit to telomere lengthening during expansion. To this end, we subjected ES cells to over 60 passages *in vitro* and analysed telomere length by quantitative fluorescence *in situ* hybridization (Q-FISH) on metaphase spreads at different passages. Mean telomere length was increased until passage 24 and then, was maintained until passage 60 (Fig. 1a and Supplementary Fig. 1a), suggesting that a few passages are sufficient to reach maximum telomere length in ES cells. This finding was further confirmed by telomere Q-FISH and telomapping techniques (Supplementary Fig. 1b,c). To discard that differences in telomere length were caused by changes in probe accessibility, or ploidy, we performed Q-FISH with a centromeric major satellite probe and found no significant differences in centromeric fluorescence at different passages (Methods, Supplementary Fig. 2). To study whether other changes might occur during *in vitro* expansion of ES cells, we subjected four independent clones of ES cells at passage 6 and passage 16 to RNA-deep sequencing and found only five genes (out of 19,555 genes analysed) significantly differentially expressed for a false discovery rate (FDR) below 0.05 analysed using DESeq (Methods). These genes were *Sox18* (upregulated 20-fold compared with ES cells at passage 6) and *Sox17*, *Zbtb48*, *Chst15* and *Jph4* (downregulated less than twofold compared with ES cells at passage 6) (Supplementary Table 1). Thus, RNA-seq analysis showed very few changes in gene expression between ES cells at passages 4 and 16. Importantly, we did not observe alterations in mRNA expression of telomerase genes, nor in components of shelterin, or in other genes associated to telomere biology. We confirmed similar telomeric repeat amplification protocol (TRAP) telomerase activity in passage 4 and passage 16 ES cells (Supplementary Fig. 3). Finally, none of the genes with altered expression levels have been implicated in longevity^36^.

Proper telomere function requires binding of the shelterin complex to telomeric repeats^1^,^5^. The shelterin protein TRF1 (telomere repeat binding factor 1) is involved in telomere capping and telomere length regulation^37^,^38^,^39^,^40^. Interestingly, TRF1 is highly expressed in ES cells as well as in induced pluripotent stem (iPS) cells^35^,^41^ and this enrichment is also maintained in adult stem cell compartments compared with more differentiated compartments in the context of the organism^41^. Thus, we next asked whether *in vitro* expansion of ES cells affected TRF1 levels. To this end, we quantified TRF1 levels at a single-cell level by immunofluorescence (IF) with anti-TRF1 antibodies using confocal microscopy (Fig. 1b,c). We found higher TRF1 levels in the ICM of the blastocyst compared with the trophectoderm or to mouse embryonic fibroblasts (Fig. 1b,c and Supplementary Fig. 4a,b). Interestingly, we found that early passage ES cells (up to passage 24) retained similarly high TRF1 levels to those present in the ICM but TRF1 levels decreased at higher passages, which may reflect a change in ES cell properties at later passages. These results indicate that maximal telomere length is achieved and high TRF1 levels are maintained after moderate *in vitro* passaging of ES cells (up to passage 24).

### ES cells with hyper-long telomeres do not show increased DNA damage

We next asked whether continuous telomere lengthening associated to *in vitro* ES cell expansion would cause DNA damage, particularly at regions difficult to replicate such as telomeres. γH2AX is a marker of double-strand breaks and dysfunctional telomeres, the latter also known as telomere damage-induced foci or telomere-induced foci (TIFs)^42^,^43^. To asses DNA damage specifically at telomeric chromatin, we performed double IF with anti-γH2AX and TRF1 antibodies to quantify TIFs (Fig. 1d–f). We found very few ES cells with DNA damage (>3 γH2AX foci per cell) up to passage 24, but this percentage significantly increased at later passages with more than 40% of the cells showing DNA damage (Fig. 1d). In the case of telomere-specific damage, we also observed low numbers of TIF-positive cells until passage 24, and this was significantly increased at later passages (Fig. 1e,f). We found similar results when we analysed telomere damage by telomere FISH combined with IF using anti 53BP1 antibody (Supplementary Fig. 4c,d). To study the specific type of DNA damage present at later passages in cells with hyper-long telomeres, we arrested cells with colcemid and performed telomere Q-FISH on metaphase spreads. We found that the only telomere aberration increased with ES cell passaging was the presence of multitelomeric signals (MTS) (Fig. 1g,h), a type of aberration previously associated to increased telomere fragility as the result of replication stress at telomeres^13^,^44^. Interestingly, TRF1, which is also decreased at later passages (Fig. 1b), has been previously shown to protect from telomere fragility^38^,^44^.

Taken together our results demonstrate that hyper-long telomeres after moderate ES cell passaging (up to 24 passages) are well capped and do not show increased DNA damage.

### Hyper-long telomeres contribute to healthy chimaeric mice

**Figure 2:**
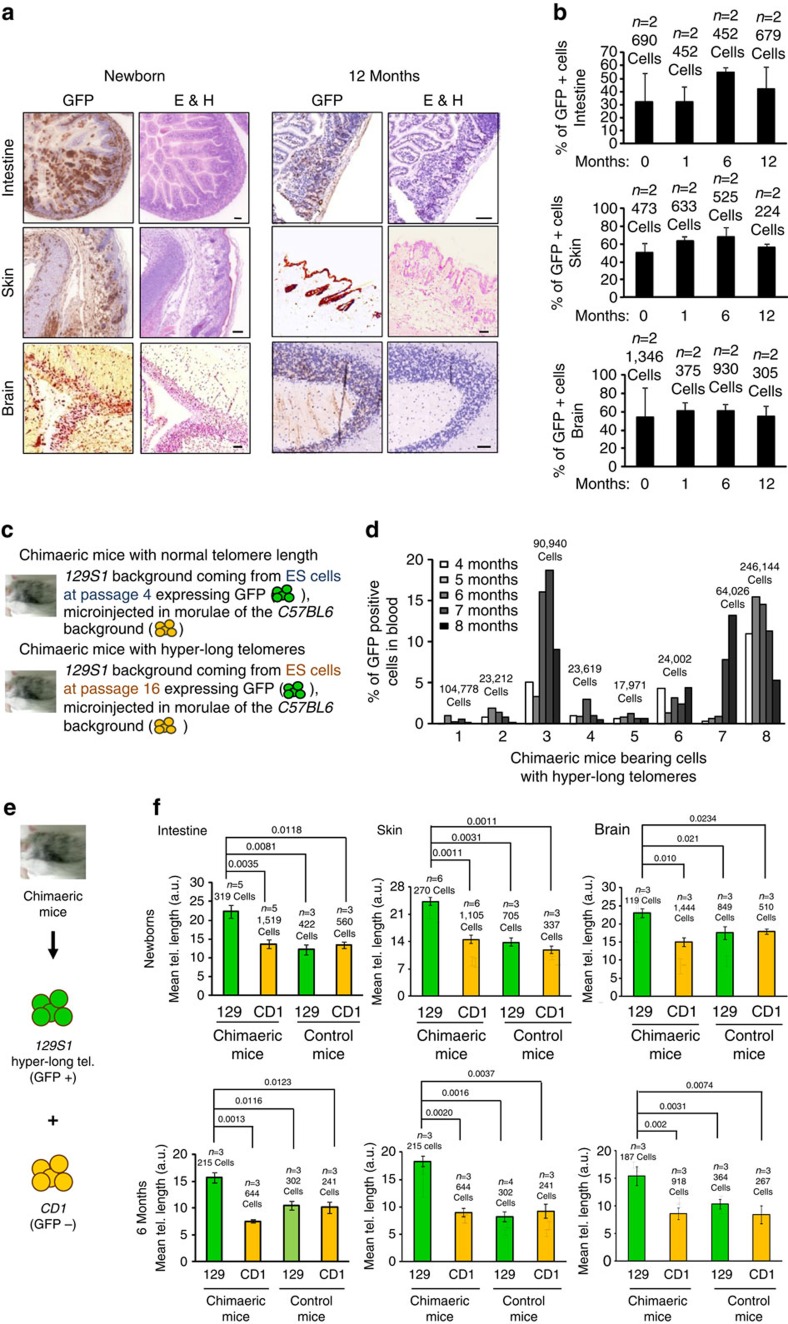
Tissues bearing cells with hyper-long telomeres are healthy. (**a**) Micrographs show histology of intestine, skin and brain from chimaeric mice bearing cells with normal telomere length and other cells with hyper-long telomeres (GFP-positive). Note that tissues containing GFP-positive cells are healthy. Scale bar, 50 μm. (**b**) The graphs show the percentage of GFP-positive cells found in intestine, brain and skin of chimaeric mice bearing hyper-long telomeres, over the period of time indicated. (**c**) The scheme shows how chimaeric mice were generated. (**d**) Per cent of GFP-positive cells in blood from chimaeric mice bearing hyper-long telomeres during the period of time is indicated. (**e**) Scheme shows that the chimaeric mice analysed were constituted by two different backgrounds, *129S1* with hyper-long telomeres and *CD1* cells with normal length telomeres. (**f**) Graphs show mean telomere length in intestine, skin and brain in newborns and 6 months chimaeric mice compared with aged-matched animals from the *129S1* and *CD1* backgrounds. Representative graph of two independent experiments. The s.e.m. was represented in error bars. Student *t*-test with the Bonferroni correction was used to calculate the *P* values. E&H, eosin and haematoxylin.

**Figure 3:**
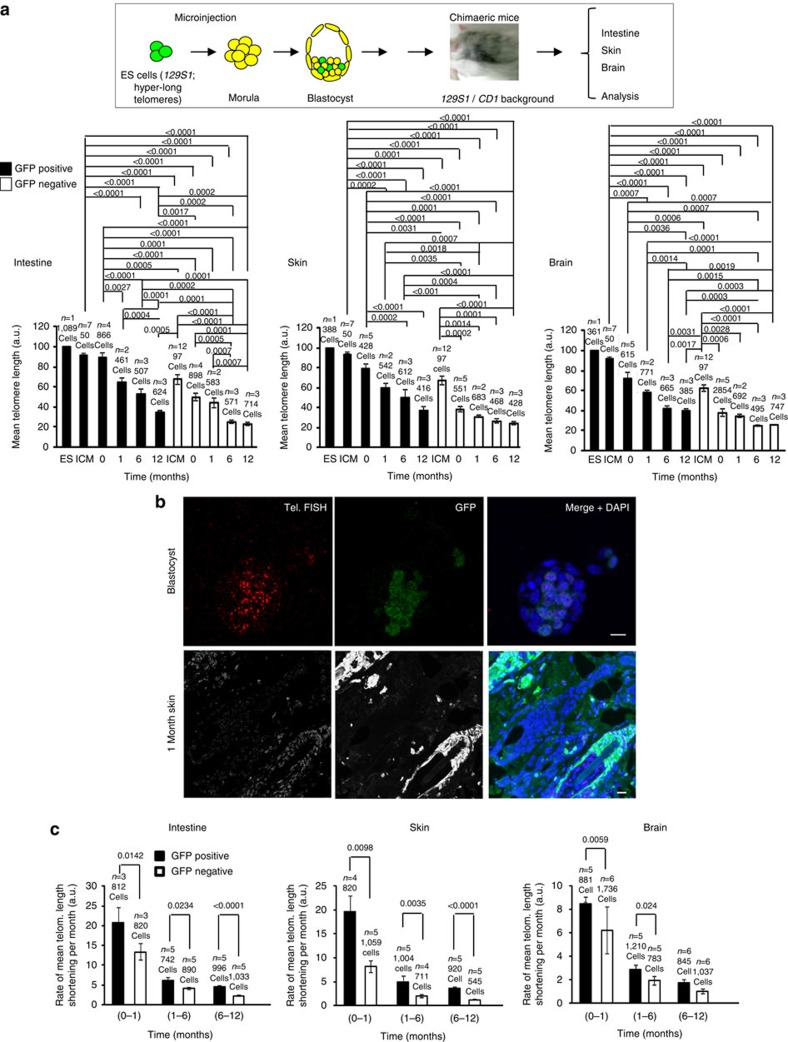
Both hyper-long and normal length telomeres shorten with age *in vivo*. (**a**) The scheme shows how the chimaeric mice were generated. Underneath, The graphs show mean telomere length in ES cells used for microinjection, The ICM of the blastocyst, intestine, skin and brain from chimaeric mice bearing hyper-long (GFP-positive) and normal length (GFP-negative) telomeres, at the indicated time points. Samples were subjected to IF with anti-GFP antibody combined with telomere FISH. Telomere length was analysed by telomapping. Representative graph of two independent experiments. (**b**) Representative images showing GFP-positive cells (green), GFP-negative cells and telomere FISH in blastocyst and in 1-month-old skin. Scale bar, 10 μm. (**c**) Graphs present the rate of mean telomere length shortening per month in intestine, skin and brain for the indicated periods. *n*=number of blastocyst, or independent clones of ES cell (passage 16) or independent chimaeric mice. Representative graph of two independent experiments. The s.e.m. was represented in error bars. Student *t*-test with the Bonferroni correction was used to calculate the *P* values.

We next addressed the capability of ES cells bearing hyper-long telomeres to contribute to chimaera formation *in vivo* and to retain hyper-long telomeres in the adult organism (Fig. 2a–f). To this end, we aggregated GFP-positive ES cells with hyper-long telomeres (passage 16) with eight-cell morulae, and derived from them chimaeric mice (see Fig. 3a; note longer telomeres in GFP-positive ICM cells compared with the GFP-negative ICM of the unmodified species). Gross phenotypic analysis of the resulting chimaeric mice showed that they were normal compared with the unmodified species. To track cells derived from GFP-positive ES cells with hyper-long telomeres, we performed immunohistochemistry of different tissues with an anti-GFP antibody. In particular, we focused our analyses in the intestine and the skin as an example of two highly proliferative tissues, as well as in the brain, as an example of a low proliferative tissue. Tissues were analysed at four time points during the mouse lifespan: 0; 1; 6; and 12 months of life. We observed the presence of both GFP-positive and -negative cells in all tissues analysed (Fig. 2a,b). The percentage of GFP-positive cells was ∼20–50 per cent in all tissues studied, and this was maintained when chimaeric mice were analysed at different time points indicating that cells bearing long telomeres are functional and maintained with ageing (Fig. 2a,b). Importantly, these chimaeric tissues showed a normal histology and we did not observe any pathological finding (Fig. 2a and Supplementary Fig. 5a). To study dynamically the fate of cells with hyper-long telomeres *in vivo*, we performed a longitudinal analysis of the percentage of cells expressing GFP in peripheral blood samples from chimaeric mice bearing either normal or hyper-long telomeres (Fig. 2d) from 4 until 8 months of age. We found that GFP-positive cells with hyper-long telomeres are maintained, or even increased in some cases, over time with respect to the starting point (Fig. 2d). As control, while GFP-positive cells containing normal telomere length were also maintained with time (Supplementary Fig. 5b). We further confirmed telomere length in GFP-positive or -negative cells by Q-FISH in blood samples from these chimaeric mice (Supplementary Fig. 6a). Note that in chimaeric mice bearing GFP-positive cells with normal telomere length both the GFP-positive or -negative cells display similar telomere intensities (upper graph and pictures), while in chimaeric mice bearing GFP-positive cells with hyper-long telomeres, GFP-positive cells display brighter telomeres than the GFP-negative cells (lower graph and pictures; Supplementary Fig. 6a,b).

Because the chimaeric mice contained two different genetic backgrounds (Fig. 2e), *129S1* cells (with hyper-long telomeres) and *CD1* cells (with normal length telomeres) from receptor morulae, we compared telomere length in the chimaeric mice with age-matched mice of the *129S1* and *CD1* backgrounds in newborns and 6-months-old chimaeric mice. As shown in Fig. 2f and Supplementary Figs 6c and 7, GFP-positive cells, bearing hyper-long telomeres had a higher mean telomere length than cells of both the *129S1* and *CD1* backgrounds with normal length telomeres. Indeed, the mean telomere length was similar in the chimaera eGFP-negative cells and in cells from non-chimaeric mice from either background (Fig. 2f).

These results demonstrate the contribution of GFP-positive ES cells with hyper-long telomeres to the formation of healthy tissues and mice which contain GFP-positive cells with longer telomeres than those of the unmodified species (GFP-negative cells). These findings suggest that cells with hyper-long telomeres are maintained during both embryonic and adult mouse development.

### Both long and normal length telomeres shorten with age

Next, we sought to investigate the length dynamics of hyper-long telomeres in the context of the organism with increasing age. To this end, we performed Q-FISH using a telomere probe to measure telomere length combined with IF using anti-GFP antibody to track the cells derived from ES cell with hyper-long telomeres in sections from the intestine, skin and brain, from chimaeric mice at 0, 1, 6 and 12 months of age (Methods). We confirmed longer telomeres in the GFP-positive ES cells used for morulae aggregation and in the derived GFP-positive ICM cells compared with the GFP-negative ICM cells within the same blastocyst (Fig. 3a,b and Supplementary Figs 8 and 9)^35^. After birth, we observed telomere shortening with age in all mouse tissues analysed, including the brain, in agreement with previous findings (Fig. 3a and Supplementary Figs 8–10)^16^. At all the different ages studied, we observed that the mean telomere length of GFP-positive cells (derived from ES cells with hyper-long telomeres) was higher than the mean telomere length of the GFP-negative cells (derived from ES cells of the unmodified species with normal length telomeres) in all tissues analysed (Fig. 3a and Supplementary Figs 8 and 9), in spite of a slightly increased rate of telomere shortening in the GFP-positive cells (Fig. 3c), probably due to the starting differences in telomere length in these cells. Note that cells with hyper-long telomeres were never found or were very rare in the GFP-negative tissue compared with the GFP-positive cells (Fig. 3a and Supplementary Figs 7–9). Interestingly, we noticed that telomere shortening with age was not uniform over time, with the highest rate of telomere shortening per month occurring during the first month of life in both the GFP-positive and -negative cells (Fig. 3c), which has also been observed in human blood^45^. This high rate of telomere shortening also affected to the brain. This fast telomere shortening soon after birth may reflect on the higher proliferation rates in newborn mice to reach the size of adults.

In summary, these findings indicate that telomere shortening occurs associated with ageing in the context of mouse tissues in both cells derived from ICM of the recipient blastocyst (GFP-negative cells) with normal length telomeres and ES cells with hyper-long telomeres (GFP-positive), and that this is exacerbated during the first month of life. Importantly, adult cells derived from GFP-positive ES cells with hyper-long telomeres showed longer telomeres at any time point in all tissues analysed indicating that we achieved generation of adult tissues bearing longer telomeres than those of the unmodified species without any genetic manipulation and in particular without transgenic germ line telomerase overexpression.

To further confirm these results, we studied chimaeric mice showing a 100% of chimaerism. In particular, we determined telomere length, shelterin levels, as well as telomerase levels in blood samples at 20 and 40 weeks after birth. We confirmed longer telomeres in the 100% GFP-positive chimaeric mice with hyper-long telomeres at both time points compared with 100% GFP-positive cells chimaeric mice with normal telomere length, as well as compared with chimaeric mice with normal telomeres and age-matched control mice (Supplementary Fig. 11a,c). In addition, 100% chimaeric mice with hyper-long telomeres showed less percentage of short telomeres compared with 100% chimaeric mice with normal telomere length (Supplementary Fig. 11a–c). We then analysed the levels of different shelterin proteins in 100% chimaeric mice with normal or hyper-long telomeres. By IF, we found similar levels of TRF1 or RAP1 shelterins in blood samples from either 100% chimaeric mice with normal or hyper-long telomeres (Supplementary Fig. 11d–f). By quantitative PCR, we also confirmed similar mRNA levels of shelterin and telomerase, both in blood and skin samples from 100% chimaeric mice with normal or hyper-long telomeres or controls (Supplementary Fig. 12). Finally, we demonstrate similar telomerase TRAP activity in spleen from chimaeric mice with normal length and hyper-long telomeres as well as in the control mice (Supplementary Fig. 13).

These results suggest that mice with hyper-long telomeres do not have globally an altered expression of shelterin or telomerase gene compared with control mice.

### Longer telomeres in adult stem and differentiated cells

**Figure 4:**
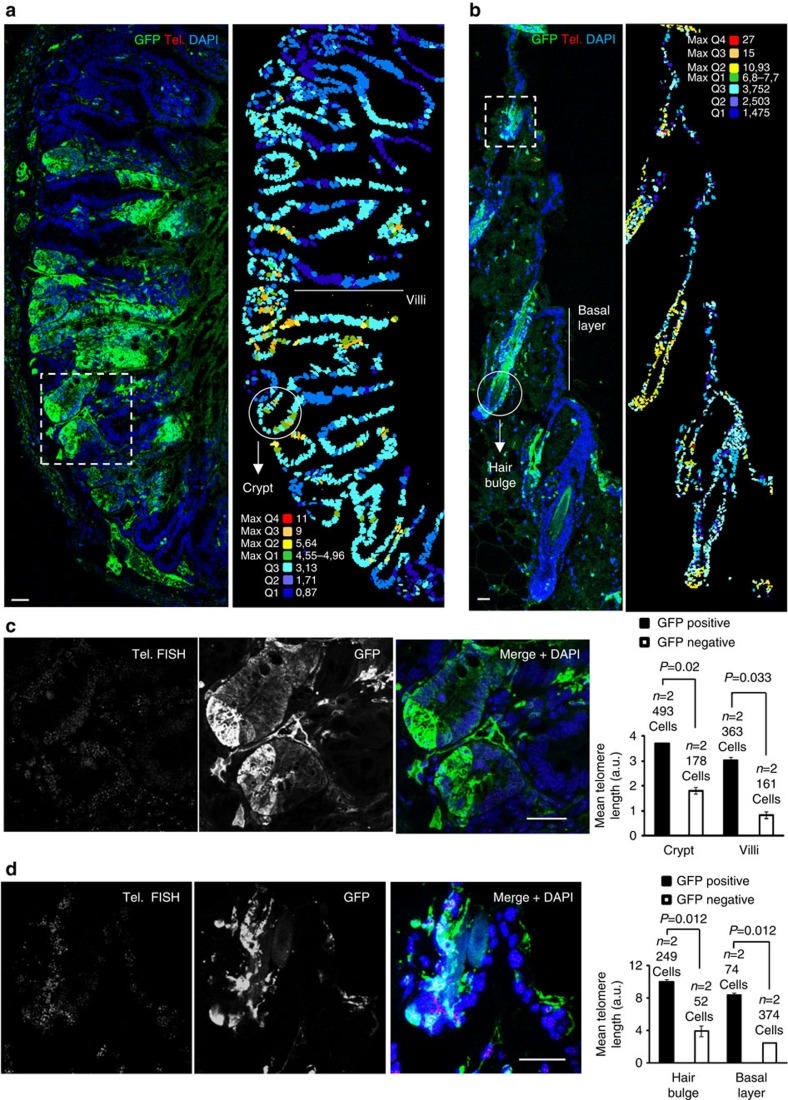
Hyper-long telomeres remain long in stem cell niches and differentiated compartments despite ageing. (**a**) Representative image of a 6 months chimaeric intestine after IF with anti-GFP antibody combined with telomere FISH (left). The map shows telomere intensity of each cell, analysed by telomapping. The colour-intensity code is specified over the image (right). (**b**) Representative image of 6 months chimaeric skin after IF with anti-GFP antibody combined with telomere FISH (left). The map shows telomere intensity for each cell, analysed by telomapping. The colour-intensity code is specified over the image (right). Below, mean telomere-length quantification of the cells contained in the stem cell niches (crypt or hair bulge) and differentiated compartments (villi and basal layer), differentiating the GFP-positive versus GFP-negative patches. Note that longer telomeres coincide with the GFP-positive patches. Representative images of five independent analysis. *n*=number of chimaeric mice. (**c**) Magnification of the square area in picture a separating the telomere FISH and the GFP signals. (**d**) Magnification of the square area in **b** separating the telomere FISH and the GFP signals. Bar, 20 μm. The s.e.m. was represented in error bars. Student *t*-test with the Bonferroni correction was used to calculate the *P* values.

Stem cell compartments are enriched in cells with the longest telomeres compared with the more differentiated compartments within the same tissue both in mice and humans^16^,^46^. Furthermore, long telomeres are advantageous for stem cell function *in vivo*^17^, as critical telomere shortening in stem cells impairs their ability to mobilize and regenerate tissues^17^ and short telomeres impair self renewal and repopulation capacity in blood and intestine^47^,^48^. Thus, we set to address whether stem cells derived from GFP-positive ES cells with hyper-long telomeres retained longer telomeres in the adult organism. In particular, we compared telomere length in GFP-positive and negative cells located at known stem cells compartments in both the skin and small intestine. Briefly, we combined IF with anti-GFP antibody with telomere Q-FISH on tissue sections to generate maps of telomere length at a single-cell level within tissues (immuno-telomapping; Methods). In the case of the intestine and skin from 6-month-old chimaeric mice (Fig. 4), GFP-positive cells located at known stem cell compartments (the intestinal crypts in the case of the small intestine and hair bulge in the case of the skin) had longer telomeres than the GFP-negative counterparts at the same compartments (Fig. 4a–d). In the case of the differentiated compartments (villi and basal layer), we also observed longer telomeres in the GFP-positive cells compared with the GFP-negative neighbouring cells (Fig. 4a–d).

Together, these results indicate that aggregation of ES cell with hyper-long telomeres results in both adult stem cell compartments and differentiated compartments containing cells with longer telomeres compared with the corresponding adult compartments in the unmodified species. This finding is in agreement with the fact that the percentage of GFP-positive cells in the tissues studied is similar in chimaeric mice of different ages.

### Lower accumulation of short telomeres with aging

**Figure 5:**
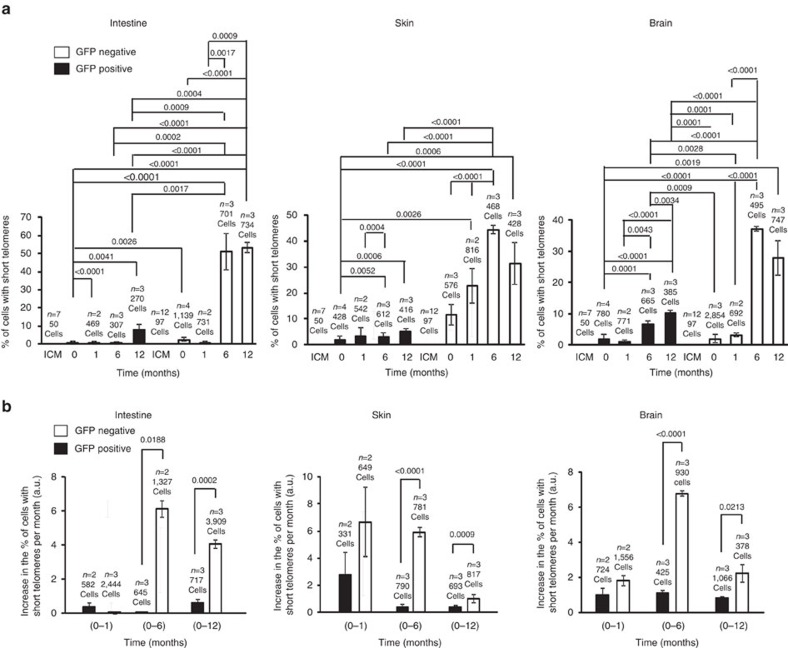
The amount of cells containing short telomeres is reduced in chimaeric mice containing hyper-long telomeres. (**a**) The graphs show the per cent of cells with short telomeres in the ICM of the blastocyst, intestine, skin and brain at the indicated time points. Samples were subjected to IF with anti-GFP antibody combined with FISH and analysed by telomapping. (**b**) Graphs show the increase in the per cent of cells with short telomeres per month in intestine, skin and brain at the indicated time points. Samples came from chimaeric mice and were analysed as described in **a**. *n*=number of blastocysts or chimaeric mice. Representative graphs of two independent experiments. The s.e.m. was represented in error bars. Student *t*-test with the Bonferroni correction was used to calculate the *P* values.

Next, we addressed whether the accumulation of cells with short telomeres with ageing was also lower in GFP-positive cells. In *M. musculus*, the 10% percentile in the reference population (in this case, the GFP-negative newborns, whose telomere length is similar to the *129S1* unmodified species in age-matched animals) is used to quantify short telomeres. Percentile 10% corresponded to a telomere length of approximately <15 kb (ref. 31). The percentage of cells with short telomeres was zero in the case of the ICM of the blastocyst for both genetic backgrounds (Fig. 5). Strikingly, the percentage of cells with short telomeres was lower for the GFP-positive cells at the different ages and tissues studied (Fig. 5a). Interestingly, tissues with a higher rate of proliferation accumulated more cells with short telomeres in both positive and negative GFP cells at the 12 months time point (Fig. 5a). In both positive and negative GFP cells, the biggest increase in the percentage of cells with short telomeres occurred during the first month of life (Fig. 5b), in agreement with a faster rate of telomere shortening early in life, until the adult organism is formed. Of note, after the first month of life, and for the rest of the time points analysed, the biggest accumulation of cells with short telomeres continued to occur in the GFP-negative cells.

Our results suggest that adult cells derived from ES cell with hyper-long telomeres preserve longer telomeres and accumulate lower numbers of short telomeres with age.

### Mice with longer telomeres show less DNA damage and tumours

**Figure 6:**
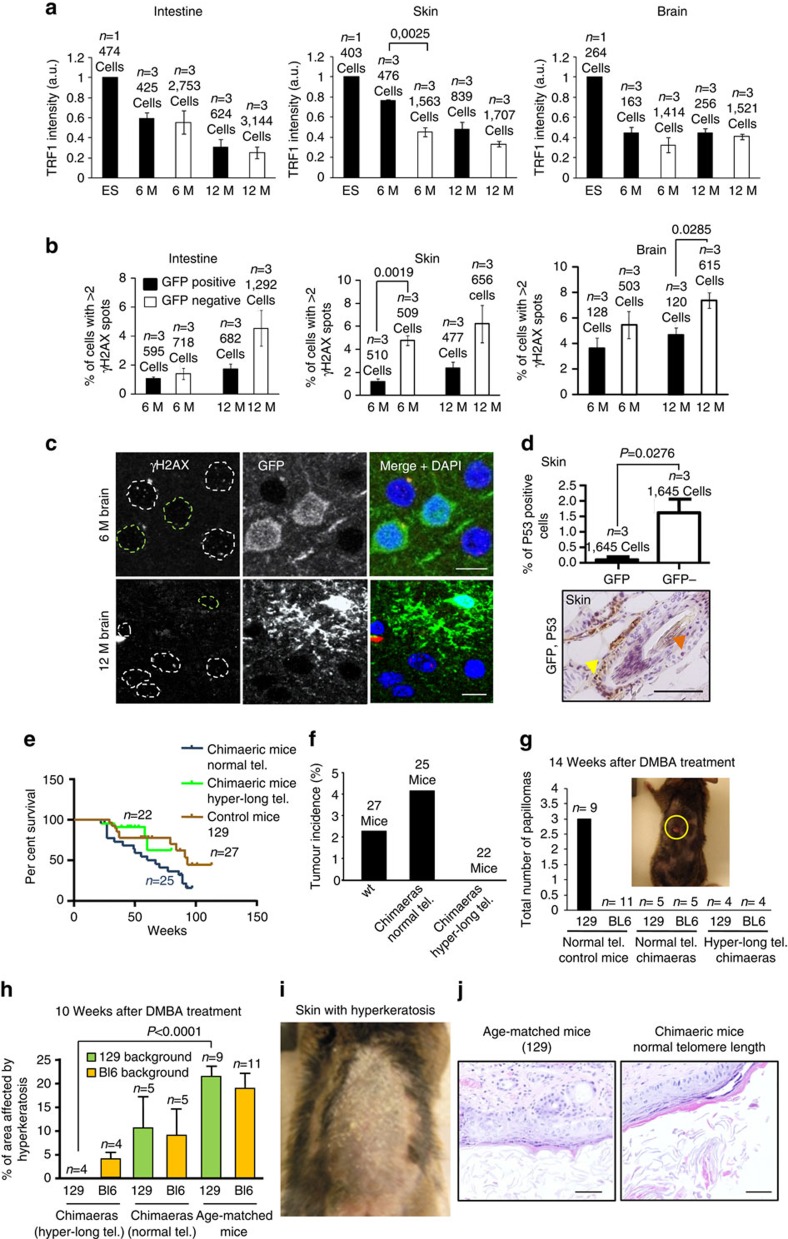
Analysis of TRF1 and DNA damage in chimaeric tissues. (**a**) Mean TRF1 intensity in cells bearing normal (GFP-negative) or hyper-long telomeres (GFP-positive) in ES cells used for the generation of chimaeric mice, and in brain, intestine and skin from the chimaeric mice at 6 and 12 months. Tissues were subjected to IF with anti-GFP and anti-TRF1 antibodies. Representative graphs of two independent experiments. (**b**) Per cent of cells positive for γH2AX in brain, intestine and skin tissue from chimaeric mice at 6 and 12 months. Tissues were subjected to IF with anti-GFP and anti-γH2AX antibodies. Representative graphs of two independent experiments. (**c**) Representative images of brain tissues as described in **b**. Scale bar, 10 μm. (**d**) The graph shows the per cent of P53-positive cells in skin from 1-year-old chimaeric mice bearing hyper-long telomeres for both GFP-positive and -negative cells. Underneath, a representative image of intestine stained for GFP and P53 and in 1-year-old chimaeric mice. The yellow arrowhead indicates GFP stain and the red arrow indicates p53 stain. Scale bar, 50 μm. (**e**) The graph shows the per cent survival of three different cohorts of mice, chimaeric mice with normal telomere length, chimaeric mice bearing cells with hyper-long telomeres and control mice. (**f**) The graphs show the percentage of spontaneous tumour incidence in the three cohorts of mice described in **e**. (**g**) Chemical carcinogenesis experiment. The graph shows the total number of papillomas in chimaeric mice bearing hyper-long telomres, normal length telomeres or in age-matched mice of the *129S1* and *C57Bl6* backgrounds. Representative graph of two independent experiments. (**h**) The graph shows the percentage of area affected by hyperkeratosis in the mice described in **g**. Representative graph of two independent experiments. (**i**) Representative micrograph of back skin of mice affected with hyperkeratosis. (**j**) E&H images on skin affected with hyperkeratosis. The lesion was diagnosed parakeratotic hyperkeratosis, as no signs of inflammation were observed. *n*=number of independent clones of ES cells or chimaeric or control mice. Scale bar, 50 μm. The s.e.m. was represented in error bars. Student *t*-test with the Bonferroni correction was used to calculate the *P* values, except for the graph of **e**, where a log rank test was used. E&H, eosin and haematoxylin.

First, as an indication of proper telomere capping, we examined TRF1 levels in both GFP-positive and -negative cells. TRF1 is an essential shelterin component that plays a role in telomere protection by preventing telomere fragility and fusions, which in turn are associated to premature tissue ageing and increased cancer susceptibility^5^,^13^,^44^,^49^. To this end, we analysed TRF1 abundance at telomeres in both ES cells used for aggregation experiments as well as in 6- and 12-moths-old chimaeric mice. A tendency to find the mean TRF1 intensity higher in the GFP-positive cells than in the GFP-negative was observed in all the tissues analysed (Fig. 6a and Supplementary Fig. 14), reflecting on adequate telomere protection.

Next, we studied accumulation of DNA damage. Long telomeres could be a target of DNA damage owing to more difficulties to replicate repetitive DNA, although DNA damage can occur independently of telomere length due to genotoxic stress^50^,^51^. We determined DNA damage by % of cells showing >2 γ-H2AX foci in tissues at 6- and 12-months-old animals (Fig. 6b,c and Supplementary Fig. 15a). We observed a moderate number of cells showing DNA damage in all the tissues analysed and this increased with ageing, but there was a tendency to accumulate more of these cells in the GFP-negative cells at both time points, in agreement with shorter telomeres in these cells. Although in this assay we cannot distinguish damage at telomeres from other regions, these findings are in line with the notion that longer telomeres are more efficiently protected from damage by maintaining a functional telomere capping structure^41^,^46^,^49^. In agreement with the lower DNA damage, we also detected increased numbers of cells positive for p53 staining in the GFP-negative population compared with the GFP-positive cells both in intestine and skin (Fig. 6d and Supplementary Fig. 15b). These results suggest that hyper-long telomeres are well capped in the context of the organism as indicated by high levels of the TRF1 protein, which in turn ensures a lower accumulation of DNA damage and of p53 in tissues with ageing.

Importantly, we found that hyper-long telomeres did not result in detectable long-term deleterious effects for mice, as indicated by a similar survival of the chimaeric mice bearing cells with hyper-long telomeres compared with chimaeric mice with normal telomere length (see Fig. 6e). In line with this, we did not find increased incidence of spontaneous tumours in the chimaeric mice with hyper-long telomeres, indeed, none of the chimaeric mice bearing hyper-long telomeres developed any spontaneous tumours in the curse of the survival follow-up (Fig. 6f). To further study whether hyper-long telomeres could be influencing cancer, we performed a 7,12-dimethylbenz[a]anthracene (DMBA) -phorbol 12-myristate 13-acetate (TPA) chemical carcinogenesis protocol on the skin of chimaeric mice with normal or hyper-long telomeres, as well as age-matched controls (Methods). DMBA was applied once on mouse skin, followed by TPA treatment during 15 weeks. The evolution of papillomas was further observed during at least 20 weeks after DMBA treatment. After 20 weeks following DMBA treatment, only *129S1* control mice showed papillomas, whereas *C57BL6* control mice and chimaeric mice with normal and hyper-long telomeres did not show papillomas (Fig. 6g and Supplementary Fig. 16). Note that the *C57Bl6* background is very resistant to papilloma formation^52^,^53^,^54^. In addition, at 10 weeks after DMBA treatment, we observed the presence of preneoplastic lesions such as parakeratotic hyperkeratosis, produced by external addition of TPA and not due to inflammation processes since neutrophils were not present in a systematic way (Fig. 6h–j) in the skin of control mice and chimaeric mice with normal telomere length. Interestingly, in chimaeric mice with hyper-long telomeres, the patches of skin with hyper-long telomeres (GFP-positive cells) did not show presence of hyperkeratosis while these lesions were readily observed in the patches of skin with normal length telomeres (GFP-negative cells) from the same mice (see Fig. 6h,i).

### Cells with hyper-long telomeres show better skin wound healing

**Figure 7:**
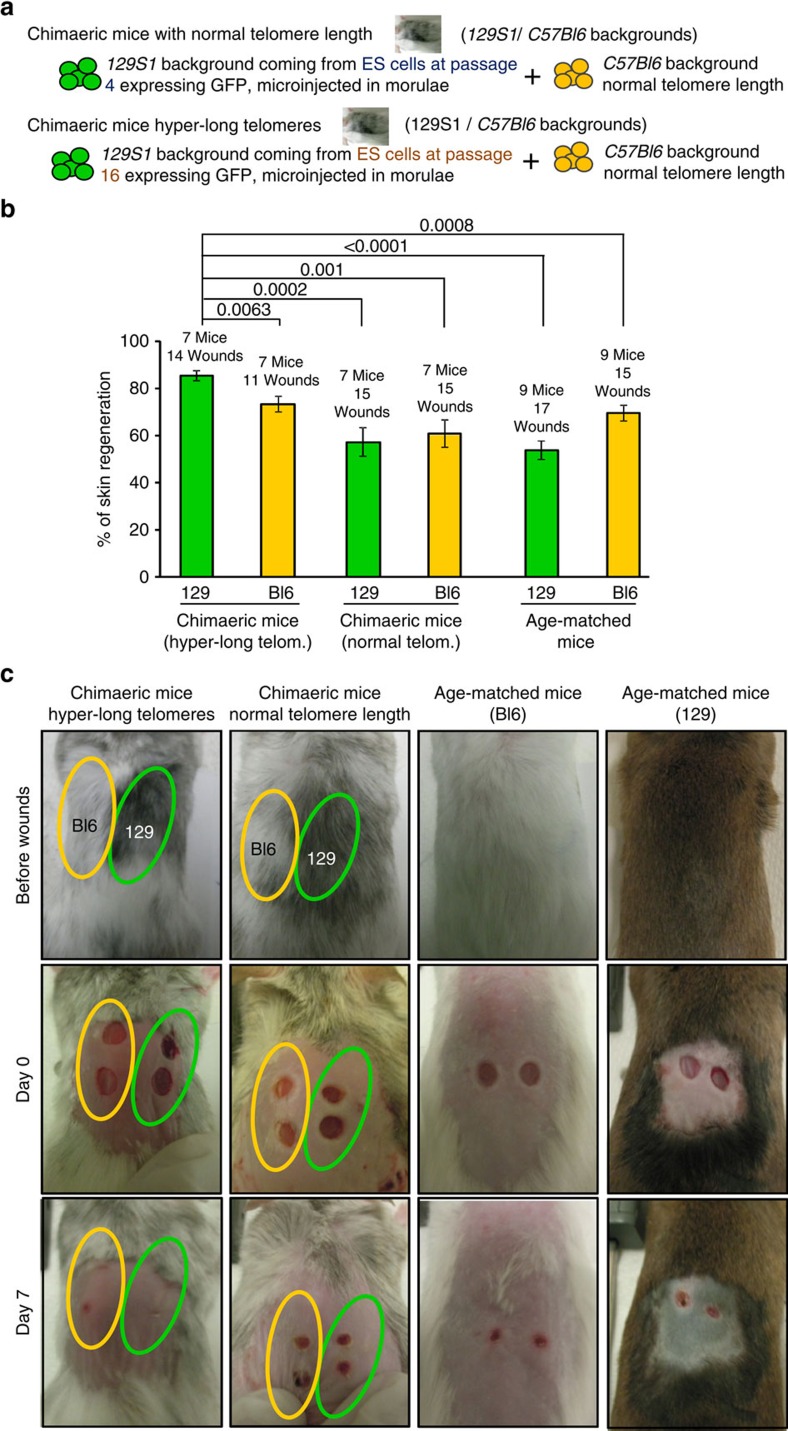
Wound-healing in chimaeric mice with normal or hyper-long telomeres. (**a**) Scheme shows that the chimaeric mice analysed were constituted by two different backgrounds, *129S1* and *C57Bl6*. While *C57Bl6* background contains cells of normal telomere length, the *129S1* cells contains cells with normal telomere length (from ES at passage 4) or cells bearing hyper-long telomeres (from ES cells at passage 16). (**b**) The graph shows the per cent of skin healed in chimaeric mice bearing normal or hyper-long telomeres and age-matched controls at 7 days after the wound on skin was performed. Wounds were made with a circular 4 mm razor blade and pictures were taken. Representative graph of two independent experiments. (**c**) Representative images of chimaeric mice (two coloured skin) and age-matched controls before and at day 7 after wounds were made. Photagraphs were taken every two days and wounds were measured using Image J software at day 0 (when wounds were performed) and at day 7. The s.e.m. was represented in error bars. Student *t*-test with the Bonferroni correction was used to calculate the *P* values.

Previous studies have shown that wild-type long telomeres have advantageous effects over short telomeres in the context of the telomerase-deficient mouse model, particularly in the ability of skin stem cells to mobilize and maintain skin homeostasis^17^ and liver regeneration^48^. Here, we wondered whether hyper-long telomeres would be advantageous compared with normal length telomeres in the context of the organism. To address this, we generated two types of chimaeric mice: chimaeric mice with normal length telomeres by microinjecting ES cells at passage 4 (telomere length being similar to telomere length of the inner cells mass of the blastocyst^35^) in morulae and expressing GFP (in order to follow these cells in chimaeric tissue), as well as chimaeric mice with hyper-long telomeres by microinjecting ES cells expressing GFP at passage 16 (with hyper-long telomeres) (see scheme in Fig. 7a). In particular, ES cells with normal or hyper-long telomeres were microinjected in recipient morulae of the *C57Bl6* background. Adult chimaeric mice bearing either normal or hyper-long telomere between 6 and 12 months and adult non-chimaeric mice of the *129S1* or *C57Bl6* backgrounds (used as age-matched controls) between 6 and 12 months were anaesthetized to remove the back hair with depilatory cream. After a period of 3 days, they were anaesthetized again to cause superficial wounds of 4 mm diameter with a circular razor blade. We quantified the surface of the 4 mm wound every day. The area of the wound was reduced with days until its closure. In chimaeric mice with normal telomere length wounds were healed at the same rate than in control non-chimaeric mice (Fig. 7b). In contrast, in chimaeric mice with hyper-long telomeres, we found that rate of wound-healing was higher in the GFP-positive cells with hyper-long telomeres compared with the GFP-negative cells and the in the non-chimaeric age-matched controls (Fig. 7b,c and Supplementary Fig. 17). Telomere length was analysed in the skin extracted from chimaeric mice with either normal or hyper-long telomeres as well as in age-matched controls when wounds were caused, confirming longer telomeres in the GFP-positive cells derived from ES cells with hyper-long telomeres (Supplementary Fig. 18). Histopathology analysis confirmed that GFP-positive cells contributed to wound-healing in the chimaeric mice with hyper-long telomeres (Supplementary Fig. 19). Together, these results indicate that hyper-long telomeres are advantageous for skin regeneration compared with normal length telomeres.

## Discussion

It has been previously shown that telomere length is a molecular determinant of ageing and lifespan in mice^22^,^55^,^56^,^57^,^58^. On one hand, short telomeres lead to accelerated ageing^22^,^24^,^26^,^47^ and longer telomeres owing to telomerase overexpression can delay ageing and increase lifespan in mice^55^,^59^. All these studies have been carried out using genetic manipulations of the telomerase genes either throughout generation of genetically modified mice or through the use of gene therapy strategies^26^,^55^,^56^,^57^,^58^,^59^. Here, we set to address the possibility of generating mice with longer telomeres than normal in the absence of any genetic manipulation.

We previously showed that telomeres lengthen during *in vitro* expansion of ES cells^35^. This telomere lengthening occurs simultaneously to the loss of heterochromatic marks at telomeres and in the absence of changes in the global DNA methylation (SINE elements) or subtelomeric methylation or telomerase activity^35^. This phenomenon is also recapitulated when differentiated cells are reprogrammed to iPS cells and expanded *in vitro*, again associated to changes at telomeric heterochromatin^13^. Of note, in the absence of telomerase differentiated cells can be reprogrammed to iPS cells, but telomeres do not lengthen^13^. These observations suggested that is possible to generate cells with hyper-long telomeres simply by *in vitro* passaging and, thus, in the absence of manipulation of the telomerase gene. However, it remained unknown whether cells derived from ES cells with hyper-long telomeres are maintained throughout embryonic and adult development in mice, and they retain longer telomeres and lower ageing-associated DNA damage in the adult organism. We first observed moderately passaged ES cells (up to passage 24) showed maximum telomere length and similarly high levels of the TRF1 protein to those characteristic of pluripotent ES cell and iPS cells^41^,^60^ as well as of cells of the *in vitro* cultured ICM^35^. Of note, excessive passaging (that is, passage 60), resulted in decreased TRF1 levels and in accumulation of telomere damage as indicated by increased presence of TIFs and MTS, a telomere aberration associated with telomere fragility and indicative of telomere replication defects^38^,^44^. These aberrations could be associated to decreased levels of TRF1 protein at late ES cell passages, as TRF1 protects cells from both types of damage^38^,^44^. Of note, in spite of decreased TRF1 protein levels at later passages, we did not observe increased telomere fusions, suggesting that a minimum threshold for telomere capping is safeguarded in ES cells. Together, these observations indicate that only a limited number of passages are necessary to generate pluripotent stem cells with hyper-long telomeres and high levels of TRF1, in the absence of telomere damage and telomere aberrations. Furthermore, a moderate number of passages ensure a very similar genome-wide expression profile between ES cells at early passages as indicated by very few gene expression changes by RNA-seq. Importantly, we did not observe any changes in the expression of telomerase, shelterin proteins, or any other genes related to telomere biology.

The main challenge in this study was the successful generation of chimaeric mice derived from ES cells with hyper-long telomeres (GFP-positive to allow lineage tracking), which contain GFP-positive cells (up to ∼50% of cells in different tissues analysed) with longer telomeres than those of the unmodified species, both at the stem cell and more differentiated compartments. Here, we obtained chimaeric mice, which show much longer telomeres than those of the unmodified species in the absence of changes in expression of telomerase or telomere-binding proteins. Importantly, chimaeric mice and tissues were healthy and did not display any histopathological alterations. Interestingly, GFP-positive cells with hyper-long telomeres maintained longer telomeres and showed a lower abundance of cells with short telomeres at different times points up to 1 year of age. In addition, GFP-positive cells showed lower levels of telomere fragility and fusions, as well as lower DNA damage with ageing. These results are in line with previous findings describing that transgenic overexpression of *TERT* in mice from a different genetic background (*C57BL6*), which also resulted in longer telomeres, also showed a lower accumulation of DNA damage with ageing^61^. Similar results were obtained by *TERT* overexpression achieved by using a gene therapy strategy^55^.

We next tested whether having hyper-long telomeres was advantageous or disadvantageous for cells in highly proliferative compartments such as the blood and the skin. Longitudinal studies in which we dynamically followed cells with hyper-long telomeres in blood with ageing, showed that these cells were maintained or even amplified over time, suggesting that hyper-long telomeres are not disadvantageous and may be positively selected. Also in line with this notion, by using skin wound-healing assays, we observed that skin cells with hyper-long telomeres were able to repair more rapidly the skin wounds compared with the skin of the unmodified species.

Whether long telomeres are protective from cancer or may increase the risk of tumour formation is still debated^61^,^62^. In our previous studies, we showed that *TERT* gene therapy using AAV9 non-integrative vectors was sufficient to delay many age-related diseases, including cancer^22^,^55^,^56^,^57^,^58^. In this regard, neither chimaeric mice with hyper-long telomeres nor control mice developed any tumour during the period of analysis, suggesting that both hyper-long and normal telomeres behave similarly with respect to tumour formation. Importantly, in line with this, we found that hyper-long telomeres did not result in any long-term deleterious effects for the mice, as indicated by a similar or slightly increased survival of the chimaeric mice bearing cells with hyper-long telomeres compared with chimaeric mice with normal telomere length. In addition, chimaeric mice with hyper-long telomeres did not demonstrate a shortened latency period for tumour formation and continued tumour-free at week 20 after the start of the treatment, although the final tumour yield is still to be concluded.

Together, these findings demonstrate that cells bearing hyper-long telomeres render juvenile telomeres *in vivo*.

These results are proof of principle that it is possible to generate adult tissues with longer telomeres than normal in the absence of genetic modifications. Future studies warrant generation of a new strain of mice with hyper-long telomeres, which will be instrumental to further study the impact of hyper-long telomeres in species longevity. Finally, our results highlight the importance of using of ES cells or iPS cells with long telomeres for regenerative medicine.

## Methods

### Cell culture conditions and blastocyst collection

Procedures for embryo collection, chimaera generation and animal experimentation were approved by CNIO-ISCIII Ethics Committee for Research and Animal Welfare (CbyBA). The two clones of *in vitro* cultured ES cells were from mice with a *C57BL6* genetic background. Chimaeric mice were the result of microinjection or aggregation of R1-eGFP ES cells (obtained from A. Nagy, The Lunenfeld-Tanenbaum Research Institute) from mice with a *129S1* genetic background in recipient morulae of the *C57BL6* or *CD1* genetic background, respectively. Primary mouse embryonic fibroblasts were obtained from 13.5-day embryos of *C57BL6* mice as described^21^. ES cells were cultured in complete KSR medium composed of DMEM (high glucose; Gibco) supplemented with serum replacement (KSR 15%, Gibco), LIF (1,000 U ml^−1^, Millipore).

Cells analysed by telomapping were pelleted, embedded in gelatin (from porcine skin type A; Sigma) to a final concentration of 5% and fixed with 10% buffered formalin overnight. Blastocysts were extracted by flushing the oviduct with M2 medium (Sigma), embedded in gelatin and fixed. Tissues from chimaeric mice were extracted and fixed with 10% buffered formalin overnight. Paraffin blocks of the fixed samples and the immunohistochemistry with anti-GFP antibodies were made by the Comparative Pathology Unit of CNIO. In particular, the Comparative Pathology Unit at CNIO, made paraffin blocks from the different mouse tissues and generated 4 mm sections, which were then deparaffinized and used for immunohistochemistry with hematoxylin and eosin (Panreac and Sigma, respectively) and with the anti-GFP antibody (Roche Diagnostics S.L.; cat. number 11814460001; 1 mg ml^−1^; dilution 1:100) according to standard procedures.

### Chimaera generation

Chimaeric mice were generated at the CNIO Transgenic Mice Unit either by microinjection of R1-eGFP ES cells into Hsd:ICR (*C57Bl6* background) morulae or by aggregation of the same ES cells with morulae (*CD1* background). Embryos were harvested from superovulated donor females at E2.5 days of gestation. For the microinjection experiments, ∼60 morulae at the eight-cell stage were microinjected with 6-10 *EGFP*-expressing R1-eGFP ES cells by laser-assisted perforation of the ‘zona pellucida'. After microinjection, embryos were incubated overnight in a drop of KSOM medium in a CO_2_ incubator at 37 °C under oil. For aggregation experiments, around 60 morulae at the eight-cell stage were aggregated with around 12 cells *EGFP*-expressing R1-eGFP ES cells by removal of the ‘zona pellucida' with Tyrodés solution (Sigma). Embryos were analysed for telomere length and eGFP fluorescence when they reached the blastocyst stage (2 days after microinjection).

### Wound-healing experiments

For the wound-healing experiments, 6–12-month-old mice (both male and female) were anaesthetized with isoflurane (IsoFlo, Esteve) and the back hair was removed with depilatory cream. After 3 days, they were anaesthetised and superficial wounds were performed with a 4 mm circular razor blade. Wounds were allowed to air-dry and photographed (a caliber was placed by the side of the mice for quantification) to follow wound-healing and measured using Image J software at the indicated times (see Supplementary Fig. 17).

### Longitudinal telomere length analysis in peripheral blood

An amount of 100 μl of blood was extracted from the facial vein of 4-month-old mice (both male and female) every following month during the next four months. Erythrocytes were lysed with Buffer EL Erythrocyte lysis buffer (Qiagen). The rest of peripheral blood cells were washed with 1 × PBS, fixed, embedded in gelatin 5% (from porcine skin type A; Sigma) and paraffin blocks were made. Sections from paraffin blocks were stained with anti-GFP antibody and the per cent of GFP-positive cells was scored by the Histopathology Unit of CNIO using Mirax Scan to digitalize the slide and Axio Vision software to quantify images.

### Skin chemical carcinogenesis assay

Age-matched (8–12-month-old, males as well as females) chimaeric mice bearing either hyper-long or normal length telomeres, as well as age-matched controls were anaesthesized (using isoflurane; Esteve) to remove 1 cm^2^ back-skin hair by using depilatory cream (Billy). A single dose of DMBA (7,12-dimethylbenz(a)anthracene] (Sigma) (100 nM; 5 μgr dissolved in 200 μl of acetone) was applied on the back skin of the mice to promote mutations which could initiate cancer as described^63^. After a week, 12.5 μg of TPA (tetradecanoyl phorbol acetate) (Sigma) dissolved in 200 μl of acetone was added two times per week during 15 weeks. The number of papillomas was determined by eye and the size was measured using a caliber and registered for each mouse on a weekly basis^63^. The evolution of papillomas was followed for at least 20 weeks after DMBA treatment.

### Quantitative FISH

ES cells were blocked in metaphase with colcemid (Gibco) for 3 h, swollen in hypotonic buffer for 10 min at 37 °C, and fixed as described in ref. 64. Metaphases were dropped on slides and Q-FISH performed as in ref. 65. TFL-Telo software^66^ was used to quantify the fluorescence intensity of telomeres from 5 to 10 metaphases for each data point. Embryos and tissues in paraffin blocks were serially sectioned (2-μm-thick sections) and rehydrated before applying Q-FISH. A telomere (Panagene) or centromere (Panagene) probe labelled with CY3 (Panagene) was used as described in ref. 65.

### Microscopy

Metaphase cells were acquired with a Leica DM 6000B Fluorescence microscope, using an objective HCX-Pl-APO 100x-1.4-0.100 as described in^35^. Sections from cells or tissues subjected to IF or Q-FISH were acquired in a confocal high-resolution microscope Leica TCS-SP5 (AOBS) as described in ref. 35. Images were acquired with the sequential mode of the microscope to avoid light bleeding into other channels. The sequential mode does two separated acquisitions, first for the GFP and secondly for the CY3 dyes (Supplementary Fig. 6b).

For co-localiztion studies, typically 10 Z-sections were acquired with excitation mercury lamp of 405 and 561 and argon source of 488 wavelengths. The detector gain for the 488 and 561 emission lights were set between 750 and 800. Co-localization was analysed in every focal plane and considered positive when the two stains green and red overlapped yielding a yellow stain.

### Telomapping of blastocysts sections

Quantitative image analysis was performed on confocal RGB images using the Definiens platform (version XD). The DAPI image was used to define the nuclear area and the Cy3 image or Alexa-488 images to quantify telomere or TRF1 fluorescence. Nuclear segmentation was calculated using the Cellenger module of Definiens software. Cy3 (telomere or centromere) or Alexa-488 (TRF1; 1:400 dilution; Life Technology A11008; lot 1408830; 2 mg ml^−1^) fluorescence intensity was measured as ‘average grey value' (total grey value per nuclei) units (arbitrary units of fluorescence). These ‘average telomere fluorescence' values represent the average Cy3 or Alexa-488 pixel intensity for the total nuclear area, therefore ruling out that differences in nuclear size may influence telomere length measurements. Telomere fluorescence values for each sample are exported to Excel to generate frequency histograms. Definiens also provides images where the nuclear mask is substituted by colours associated to CY3 intensities, generating tissue-specific maps of telomere length.

### IF combined with FISH

Antigen retrieval of the paraffin sections were done by the Comparative Pathology Unit of CNIO. Samples were permeabilized with 0.5% Triton X-100 for 1.5 h at room temperature. Cells were blocked in PBS with 5% BSA and incubated overnight at 4°C with antibodies against GFP (1:500 dilution; Roche Diagnostics S.L.; cat. number 11814460001; 1 mg ml^−1^; dilution 1:400), TRF1 (Abcam, cat. number Ab10579; lot 598474; dilution 1:100), anti-γH2AX (Millipore, cat. number 05-636; lot 2138016; 1 mg ml^−1^; dilution 1:400), anti 53BP1 (Millipore, cat. number MAB3804; lot 1999450; 10 mg ml^−1^; dilution 1:400), anti Rap1 antibody (Bethyl Laboratories, ING; A300-306-A; 1 mg ml^−1^; dilution 1:400) and rabbit anti-TRF1 generated in the laboratory (1:400 dilution; lot 226) (ref. 67). Secondary antibodies (Alexa Fluor 488 rabbit 1:400 dilution; Life Technology A11008; lot 1408830; 2 mg ml^−1^) were incubated at room temperature for 45 minutes. Samples were fixed in 4% formaldehyde, dehydrated and incubated with a telomere probe labelled with CY3 (Panagene) as described in ref. 65.

### RNA sequencing

One microgram of total RNA per sample was used. Sample RNA Integrity Numbers were 10 when assayed on an Agilent 2100 Bioanalyzer. PolyA+ fraction was purified and randomly fragmented, converted to double stranded cDNA and processed through subsequent enzymatic treatments of end-repair, dA-tailing, and ligation to adapters as in Illumina's ‘TruSeq Stranded mRNA Sample Preparation Part # 15031047 Rev. D' kit (this kit incorporates dUTP during second strand cDNA synthesis, which implies that only the cDNA strand generated during first strand synthesis is eventually sequenced). Adapter-ligated library was completed by PCR with Illumina PE primers (eight cycles). The resulting purified cDNA library was applied to an Illumina flow cell for cluster generation and sequenced on an Illumina instrument (see below) by following manufacturer's protocols.

Fastq^68^ files with 50-nt single-end sequenced reads were quality-checked with FastQC (S. Andrews, http://www.bioinformatics.babraham.ac.uk/projects/fastqc/) and aligned to the mouse genome (GRCm38/mm10) with TopHat-2.0.10 (ref. 69), using Bowtie 1.0.0 (ref. 70) and Samtools 0.1.19 (ref. 71), allowing two mismatches and five multihits. Raw counts for genes were obtained with HTSeq v0.5.3p9 (ref. 72), using the mouse genome annotation data set GRCm38/mm10 from the UCSC Genome Browser^73^. Differential expression was obtained with DESeq2 (ref. 74). Differences were considered significant when FDR-adjusted *P* values were lower than 0.05 FDR.

### Quantitative real-time PCR (qPCR)

Total RNA was extracted from blood samples free of erythrocytes with TRIzol (Invitrogen). The RNA in ethanol was further purified with the RNAeasy Mini kit (QIAGEN) for RNA elution, following the manufacturer's instructions.

Total RNA of 300 mg of tail skin Skin was preextracted with the kit RNaesy Fibrous Tissue Mini Kit (QUIAGEN) and further purified with the RNAeasy Mini kit (QIAGEN) following manufacturer's instructions. Up to 1 μg of RNA was used to synthesize complementary DNA with the iScript cDNA Synthesis Kit (BIO-RAD) according to manufacturer's instructions. The levels of gene transcription were determined using the primers for shelterins or telomerase (described below) using the Power SYBR Green PCR Master mix (Applied Biosystems). The PCR was carried out in a Quantstudio 6 Flex Real Time system (Applied Biosystems). Primers used are in Supplementary Table 2.

### Telomerase activity TRAP assays

ES cells were trypsinized and washed in PBS, spleen was washed in PBS after isolation, and then S-100 extracts were prepared as described in ref. 21. Three protein concentrations were used for each sample (5, 2.5 and 1 μg) for the ES cells, and two protein concentrations were used for each sample (5 and 1 μg) of spleen. Extension and amplification reactions and electrophoresis were performed as described in ref. 21. A negative control was included by preincubating each splenn extract with RNase for 10 min at 30 °C before the extension reaction and internal control for PCR efficiency was also included.

### Statistical analysis

All statistical analyses in this study were performed using the GraphPad Prism software version 5. Mean values reflect the arithmetic mean. Mean values and the s.e.m. were calculated according to the number of clones or mice used. Student's *t*-test with ‘two tails' was used to obtain the *P* value. To avoid errors due to the multiple comparison problem we have applied the Bonferroni correction to all Student's *t*-test analysis.

A log rank test was used to calculate statistical differences in survival of the different mouse cohorts. The number of mice used for the different experiments is indicated in the Figures.

### Data availability

RNA-seq gene expression data have been deposited in GEO under accession code GSE76837. The authors declare that all other data supporting the findings of this study are available within the article and its Supplementary Information file.

## Additional information

**How to cite this article**: Varela, E. *et al*. Generation of mice with longer and better preserved telomeres in the absence of genetic manipulations. *Nat. Commun.* 7:11739 doi: 10.1038/ncomms11739 (2016).

## Supplementary Material

Supplementary Information Supplementary Figures 1-19, Supplementary Tables 1-2 and Supplementary References.

## References

[b1] de Lange T. Shelterin: the protein complex that shapes and safeguards human telomeres. Genes Dev. 19, 2100–2110 (2005). 16166375 10.1101/gad.1346005

[b2] Blackburn E. H. Structure and function of telomeres. Nature 350, 569–573 (1991). 1708110 10.1038/350569a0

[b3] Blasco M. A. Telomeres and human disease: ageing, cancer and beyond. Nat. Rev. Genet. 6, 611–622 (2005). 16136653 10.1038/nrg1656

[b4] Chan S. W. & Blackburn E. H. New ways not to make ends meet: telomerase, DNA damage proteins and heterochromatin. Oncogene 21, 553–563 (2002). 11850780 10.1038/sj.onc.1205082

[b5] Martinez P. & Blasco M. A. Telomeric and extra-telomeric roles for telomerase and the telomere-binding proteins. Nat. Rev. Cancer 11, 161–176 (2011). 21346783 10.1038/nrc3025

[b6] Olovnikov A. M. A theory of marginotomy. The incomplete copying of template margin in enzymic synthesis of polynucleotides and biological significance of the phenomenon. J. Theor. Biol. 41, 181–190 (1973). 4754905 10.1016/0022-5193(73)90198-7

[b7] Olovnikov A. M. Principle of marginotomy in template synthesis of polynucleotides. Dokl. Akad. Nauk SSSR 201, 1496–1499 (1971). 5158754

[b8] Greider C. W. & Blackburn E. H. The telomere terminal transferase of Tetrahymena is a ribonucleoprotein enzyme with two kinds of primer specificity. Cell 51, 887–898 (1987). 3319189 10.1016/0092-8674(87)90576-9

[b9] Greider C. W. & Blackburn E. H. Identification of a specific telomere terminal transferase activity in Tetrahymena extracts. Cell 43, 405–413 (1985). 3907856 10.1016/0092-8674(85)90170-9

[b10] Armanios M. & Greider C. W. Telomerase and cancer stem cells. Cold Spring Harb. Symp. Quant. Biol. 70, 205–208 (2005). 16869755 10.1101/sqb.2005.70.030

[b11] Blackburn E. H., Greider C. W. & Szostak J. W. Telomeres and telomerase: the path from maize, Tetrahymena and yeast to human cancer and aging. Nat. Med. 12, 1133–1138 (2006). 17024208 10.1038/nm1006-1133

[b12] Blasco M. A. Telomere length, stem cells and aging. Nat. Chem. Biol. 3, 640–649 (2007). 17876321 10.1038/nchembio.2007.38

[b13] Marion R. M. . Telomeres acquire embryonic stem cell characteristics in induced pluripotent stem cells. Cell Stem Cell 4, 141–154 (2009). 19200803 10.1016/j.stem.2008.12.010

[b14] Marion R. M. & Blasco M. A. Telomeres and telomerase in adult stem cells and pluripotent embryonic stem cells. Adv. Exp. Med. Biol. 695, 118–131 (2010). 21222203 10.1007/978-1-4419-7037-4_9

[b15] Harley C. B., Futcher A. B. & Greider C. W. Telomeres shorten during ageing of human fibroblasts. Nature 345, 458–460 (1990). 2342578 10.1038/345458a0

[b16] Flores I. . The longest telomeres: a general signature of adult stem cell compartments. Genes Dev. 22, 654–667 (2008). 18283121 10.1101/gad.451008 PMC2259034

[b17] Flores I., Cayuela M. L. & Blasco M. A. Effects of telomerase and telomere length on epidermal stem cell behavior. Science 309, 1253–1256 (2005). 16037417 10.1126/science.1115025

[b18] Collado M., Blasco M. A. & Serrano M. Cellular senescence in cancer and aging. Cell 130, 223–233 (2007). 17662938 10.1016/j.cell.2007.07.003

[b19] Deng Y., Chan S. S. & Chang S. Telomere dysfunction and tumour suppression: the senescence connection. Nat. Rev. Cancer 8, 450–458 (2008). 18500246 10.1038/nrc2393 PMC3688269

[b20] Armanios M. Y. . Telomerase mutations in families with idiopathic pulmonary fibrosis. N. Engl. J. Med. 356, 1317–1326 (2007). 17392301 10.1056/NEJMoa066157

[b21] Blasco M. A. . Telomere shortening and tumor formation by mouse cells lacking telomerase RNA. Cell 91, 25–34 (1997). 9335332 10.1016/s0092-8674(01)80006-4

[b22] Hemann M. T., Strong M.A., Hao L.Y. & Greider C.W. The shortest telomere, not average telomere length, is critical for cell viability and chromosome stability. Cell 107, 67–77 (2001). 11595186 10.1016/s0092-8674(01)00504-9

[b23] Kong C. M., Lee X. W. & Wang X. Telomere shortening in human diseases. FEBS J. 280, 3180–3193 (2013). 23647631 10.1111/febs.12326

[b24] Lee H. W. . Essential role of mouse telomerase in highly proliferative organs. Nature 392, 569–574 (1998). 9560153 10.1038/33345

[b25] Yamaguchi H. Mutations of telomerase complex genes linked to bone marrow failures. J. Nippon Med. Sch. 74, 202–209 (2007). 17625368 10.1272/jnms.74.202

[b26] Blasco M. A. . Mouse models for the study of telomerase. Ciba Found. Symp. 211, 160–170 discussion 170-166 (1997). 9524757 10.1002/9780470515433.ch11

[b27] Vulliamy T. J. . Mutations in dyskeratosis congenita: their impact on telomere length and the diversity of clinical presentation. Blood 107, 2680–2685 (2006). 16332973 10.1182/blood-2005-07-2622

[b28] Gardner J. P. . Telomere dynamics in macaques and humans. J. Gerontol. A Biol. Sci. Med. Sci. 62, 367–374 (2007). 17452729 10.1093/gerona/62.4.367

[b29] Gomes N. M. . Comparative biology of mammalian telomeres: hypotheses on ancestral states and the roles of telomeres in longevity determination. Aging Cell 10, 761–768 (2011). 21518243 10.1111/j.1474-9726.2011.00718.x PMC3387546

[b30] Gomes N. M., Shay J. W. & Wright W. E. Telomere biology in Metazoa. FEBS Lett. 584, 3741–3751 (2010). 20655915 10.1016/j.febslet.2010.07.031 PMC2928394

[b31] Vera E., Bernardes de Jesus B., Foronda M., Flores J. M. & Blasco M. A. The rate of increase of short telomeres predicts longevity in mammals. Cell Rep. 2, 732–737 (2012). 23022483 10.1016/j.celrep.2012.08.023

[b32] Fick L. J. . Telomere length correlates with life span of dog breeds. Cell Rep. 2, 1530–1536 (2012). 23260664 10.1016/j.celrep.2012.11.021

[b33] Heidinger B. J. . Telomere length in early life predicts lifespan. Proc. Natl Acad. Sci. USA 109, 1743–1748 (2012). 22232671 10.1073/pnas.1113306109 PMC3277142

[b34] Schaetzlein S. . Telomere length is reset during early mammalian embryogenesis. Proc. Natl Acad. Sci. USA 101, 8034–8038 (2004). 15148368 10.1073/pnas.0402400101 PMC419552

[b35] Varela E., Schneider R. P., Ortega S. & Blasco M. A. Different telomere-length dynamics at the inner cell mass versus established embryonic stem (ES) cells. Proc. Natl Acad. Sci. USA 108, 15207–15212 (2011). 21873233 10.1073/pnas.1105414108 PMC3174656

[b36] Scott C. T. & DeFrancesco L. Selling long life. Nat. Biotechnol. 33, 31–40 (2015). 25574633 10.1038/nbt.3108

[b37] Ancelin K. . Targeting assay to study the cis functions of human telomeric proteins: evidence for inhibition of telomerase by TRF1 and for activation of telomere degradation by TRF2. Mol. Cell. Biol. 22, 3474–3487 (2002). 11971978 10.1128/MCB.22.10.3474-3487.2002 PMC133804

[b38] Martinez P. . Increased telomere fragility and fusions resulting from TRF1 deficiency lead to degenerative pathologies and increased cancer in mice. Genes Dev. 23, 2060–2075 (2009). 19679647 10.1101/gad.543509 PMC2751970

[b39] Smogorzewska A. . Control of human telomere length by TRF1 and TRF2. Mol. Cell. Biol. 20, 1659–1668 (2000). 10669743 10.1128/mcb.20.5.1659-1668.2000 PMC85349

[b40] van Steensel B. & de Lange T. Control of telomere length by the human telomeric protein TRF1. Nature 385, 740–743 (1997). 9034193 10.1038/385740a0

[b41] Schneider R. P. . TRF1 is a stem cell marker and is essential for the generation of induced pluripotent stem cells. Nat. Commun. 4, 1946 (2013). 23735977 10.1038/ncomms2946

[b42] d'Adda di Fagagna F. . A DNA damage checkpoint response in telomere-initiated senescence. Nature 426, 194–198 (2003). 14608368 10.1038/nature02118

[b43] Takai H., Smogorzewska A. & de Lange T. DNA damage foci at dysfunctional telomeres. Curr. Biol. 13, 1549–1556 (2003). 12956959 10.1016/s0960-9822(03)00542-6

[b44] Sfeir A. . Mammalian telomeres resemble fragile sites and require TRF1 for efficient replication. Cell 138, 90–103 (2009). 19596237 10.1016/j.cell.2009.06.021 PMC2723738

[b45] Holmes D. K. . Telomere length dynamics differ in foetal and early post-natal human leukocytes in a longitudinal study. Biogerontology 10, 279–284 (2009). 18989747 10.1007/s10522-008-9194-y

[b46] Marion R. M. & Blasco M. A. Telomere rejuvenation during nuclear reprogramming. Curr. Opin. Genet. Dev. 20, 190–196 (2009). 20176474 10.1016/j.gde.2010.01.005

[b47] Hao L. Y. . Short telomeres, even in the presence of telomerase, limit tissue renewal capacity. Cell 123, 1121–1131 (2005). 16360040 10.1016/j.cell.2005.11.020

[b48] Satyanarayana A. . Telomere shortening impairs organ regeneration by inhibiting cell cycle re-entry of a subpopulation of cells. EMBO J. 22, 4003–4013 (2003). 12881434 10.1093/emboj/cdg367 PMC169040

[b49] de Lange T. How telomeres solve the end-protection problem. Science 326, 948–952 (2009). 19965504 10.1126/science.1170633 PMC2819049

[b50] Fumagalli M. . Telomeric DNA damage is irreparable and causes persistent DNA-damage-response activation. Nat. Cell. Biol. 14, 355–365 (2012). 22426077 10.1038/ncb2466 PMC3717580

[b51] Hewitt G. . Telomeres are favoured targets of a persistent DNA damage response in ageing and stress-induced senescence. Nat. Commun. 3, 708 (2011). 22426229 10.1038/ncomms1708 PMC3292717

[b52] Blanco R., Munoz P., Flores J. M., Klatt P. & Blasco M. A. Telomerase abrogation dramatically accelerates TRF2-induced epithelial carcinogenesis. Genes Dev. 21, 206–220 (2007). 17234886 10.1101/gad.406207 PMC1770903

[b53] Munoz P. . TRF1 controls telomere length and mitotic fidelity in epithelial homeostasis. Mol. Cell. Biol. 29, 1608–1625 (2009). 19124610 10.1128/MCB.01339-08 PMC2648247

[b54] Munoz P., Blanco R., Flores J. M. & Blasco M. A. XPF nuclease-dependent telomere loss and increased DNA damage in mice overexpressing TRF2 result in premature aging and cancer. Nat. Genet. 37, 1063–1071 (2005). 16142233 10.1038/ng1633

[b55] Bernardes de Jesus B. . Telomerase gene therapy in adult and old mice delays aging and increases longevity without increasing cancer. EMBO Mol. Med. 4, 691–704 (2012). 22585399 10.1002/emmm.201200245 PMC3494070

[b56] Gonzalez-Suarez E., Geserick C., Flores J. M. & Blasco M. A. Antagonistic effects of telomerase on cancer and aging in K5-mTert transgenic mice. Oncogene 24, 2256–2270 (2005). 15688016 10.1038/sj.onc.1208413

[b57] Gonzalez-Suarez E. . Increased epidermal tumors and increased skin wound healing in transgenic mice overexpressing the catalytic subunit of telomerase, mTERT, in basal keratinocytes. EMBO J. 20, 2619–2630 (2001). 11387197 10.1093/emboj/20.11.2619 PMC125492

[b58] Jaskelioff M. . Telomerase reactivation reverses tissue degeneration in aged telomerase-deficient mice. Nature 469, 102–106 (2011). 21113150 10.1038/nature09603 PMC3057569

[b59] Tomas-Loba A. . Telomerase reverse transcriptase delays aging in cancer-resistant mice. Cell 135, 609–622 (2008). 19013273 10.1016/j.cell.2008.09.034

[b60] Boue S., Paramonov I., Barrero M. J. & Izpisua Belmonte J. C. Analysis of human and mouse reprogramming of somatic cells to induced pluripotent stem cells. What is in the plate? PLoS ONE 5, e12664 (2010). 20862250 10.1371/journal.pone.0012664 PMC2941458

[b61] Hou L., Zhang X., Gawron A. J. & Liu J. Surrogate tissue telomere length and cancer risk: shorter or longer? Cancer Lett. 319, 130–135 (2012). 22269209 10.1016/j.canlet.2012.01.028

[b62] Martinez-Delgado B. . Short telomeres are frequent in hereditary breast tumors and are associated with high tumor grade. Breast Cancer Res. Treat. 141, 231–242 (2013). 24036693 10.1007/s10549-013-2696-6

[b63] Palla A. R. . The pluripotency factor NANOG promotes the formation of squamous cell carcinomas. Sci. Rep. 5, 10205 (2015). 25988972 10.1038/srep10205 PMC4437308

[b64] Samper E., Flores J. M. & Blasco M. A. Restoration of telomerase activity rescues chromosomal instability and premature aging in Terc-/- mice with short telomeres. EMBO Rep. 2, 800–807 (2001). 11520856 10.1093/embo-reports/kve174 PMC1084029

[b65] Gonzalo S. . DNA methyltransferases control telomere length and telomere recombination in mammalian cells. Nat. Cell Biol. 8, 416–424 (2006). 16565708 10.1038/ncb1386

[b66] Zijlmans J. M. . Telomeres in the mouse have large inter-chromosomal variations in the number of T2AG3 repeats. Proc. Natl Acad. Sci. USA 94, 7423–7428 (1997). 9207107 10.1073/pnas.94.14.7423 PMC23837

[b67] Povedano J. M., Martinez P., Flores J. M., Mulero F. & Blasco M. A. Mice with pulmonary fibrosis driven by telomere dysfunction. Cell Rep. 12, 286–299 (2014). 26146081 10.1016/j.celrep.2015.06.028

[b68] Cock P. J., Fields C. J., Goto N., Heuer M. L. & Rice P. M. The Sanger FASTQ file format for sequences with quality scores, and the Solexa/Illumina FASTQ variants. Nucleic Acids Res. 38, 1767–1771 (2010). 20015970 10.1093/nar/gkp1137 PMC2847217

[b69] Trapnell C. . Differential gene and transcript expression analysis of RNA-seq experiments with TopHat and Cufflinks. Nat. Protoc. 7, 562–578 (2012). 22383036 10.1038/nprot.2012.016 PMC3334321

[b70] Langmead B., Trapnell C., Pop M. & Salzberg S.L. Ultrafast and memory-efficient alignment of short DNA sequences to the human genome. Genome Biol. 10, R25 (2009). 19261174 10.1186/gb-2009-10-3-r25 PMC2690996

[b71] Li H. . The Sequence Alignment/Map format and SAMtools. Bioinformatics 25, 2078–2079 (2009). 19505943 10.1093/bioinformatics/btp352 PMC2723002

[b72] Anders S., Pyl P. T. & Huber W. HTSeq--a Python framework to work with high-throughput sequencing data. Bioinformatics 31, 166–169 (2015). 25260700 10.1093/bioinformatics/btu638 PMC4287950

[b73] Karolchik D. . The UCSC Genome Browser database: 2014 update. Nucleic Acids Res. 42, D764–D770 (2014). 24270787 10.1093/nar/gkt1168 PMC3964947

[b74] Love M. I., Huber W. & Anders S. Moderated estimation of fold change and dispersion for RNA-seq data with DESeq2. Genome Biol. 15, 550 (2014). 25516281 10.1186/s13059-014-0550-8 PMC4302049

