# Supplementary material for: Generation of mice with longer and better preserved telomeres in the absence of genetic manipulations
Source: Nat Commun. 2016 Jun 2;7:11739. doi: 10.1038/ncomms11739 (PMC4895768; doi:10.1038/ncomms11739)
Supplement: Supplementary Information — Supplementary Figures 1-19, Supplementary Tables 1-2 and Supplementary References. [file ncomms11739-s1.pdf]

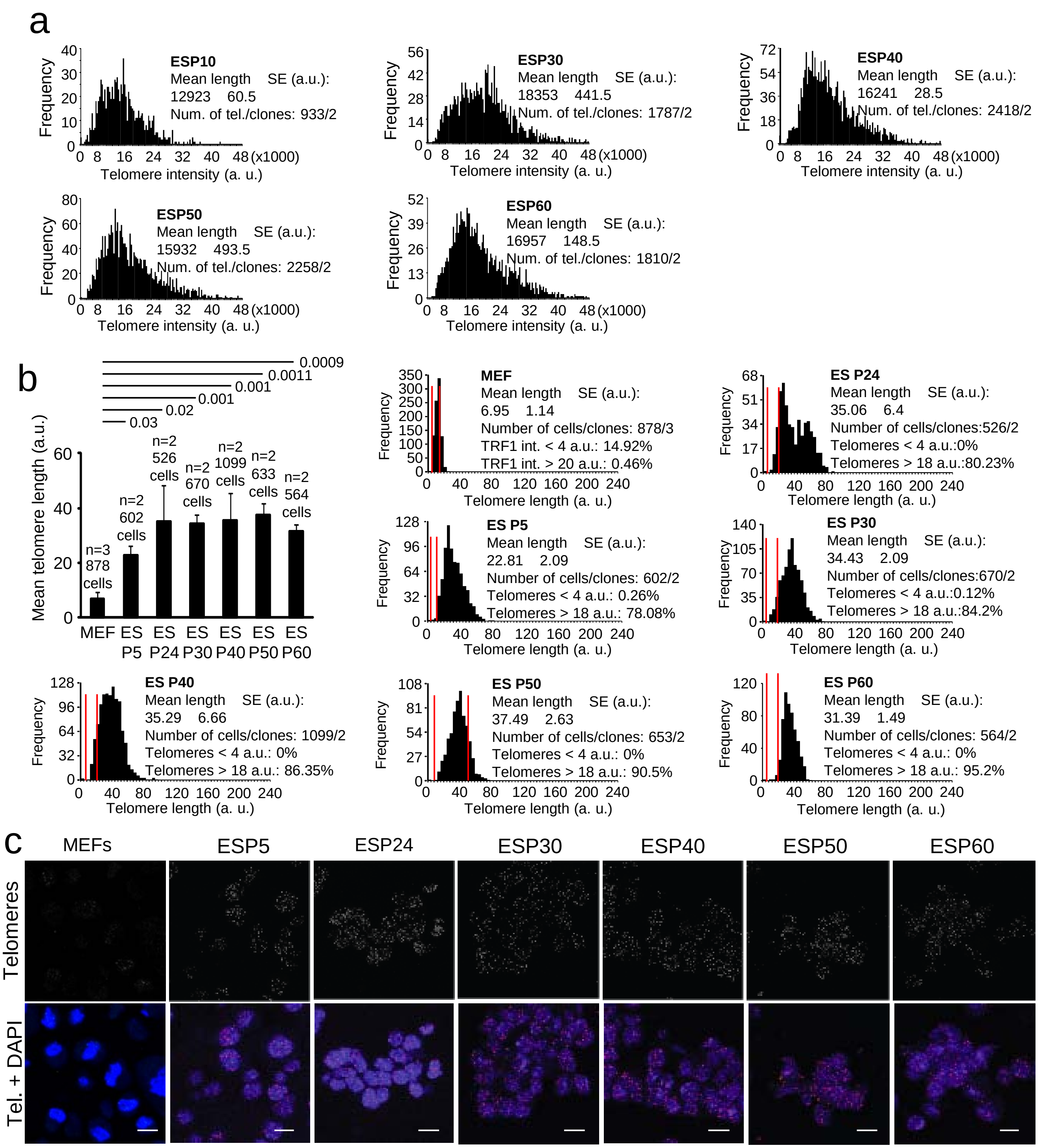

Supplementary Figure 1. Telomere Q-FISH in MEFs, blastocysts, and ESCs. a) Histograms correspond to telomere length from ESCs at different passages arrested in metaphase with colcemid. b) Mean telomere length of primary MEFs (passage 2) and ESCs at the indicated passages, embedded in paraffin and analyzed by telomapping. Histograms correspond to telomere intensity in the samples described above. c) Representative images of telomere FISH in the samples described in b. Scale bar: 10µm. Student T-test with the Bonferroni correction was used to calculate the p-values.

Varela et al., Supplementary Figure 1

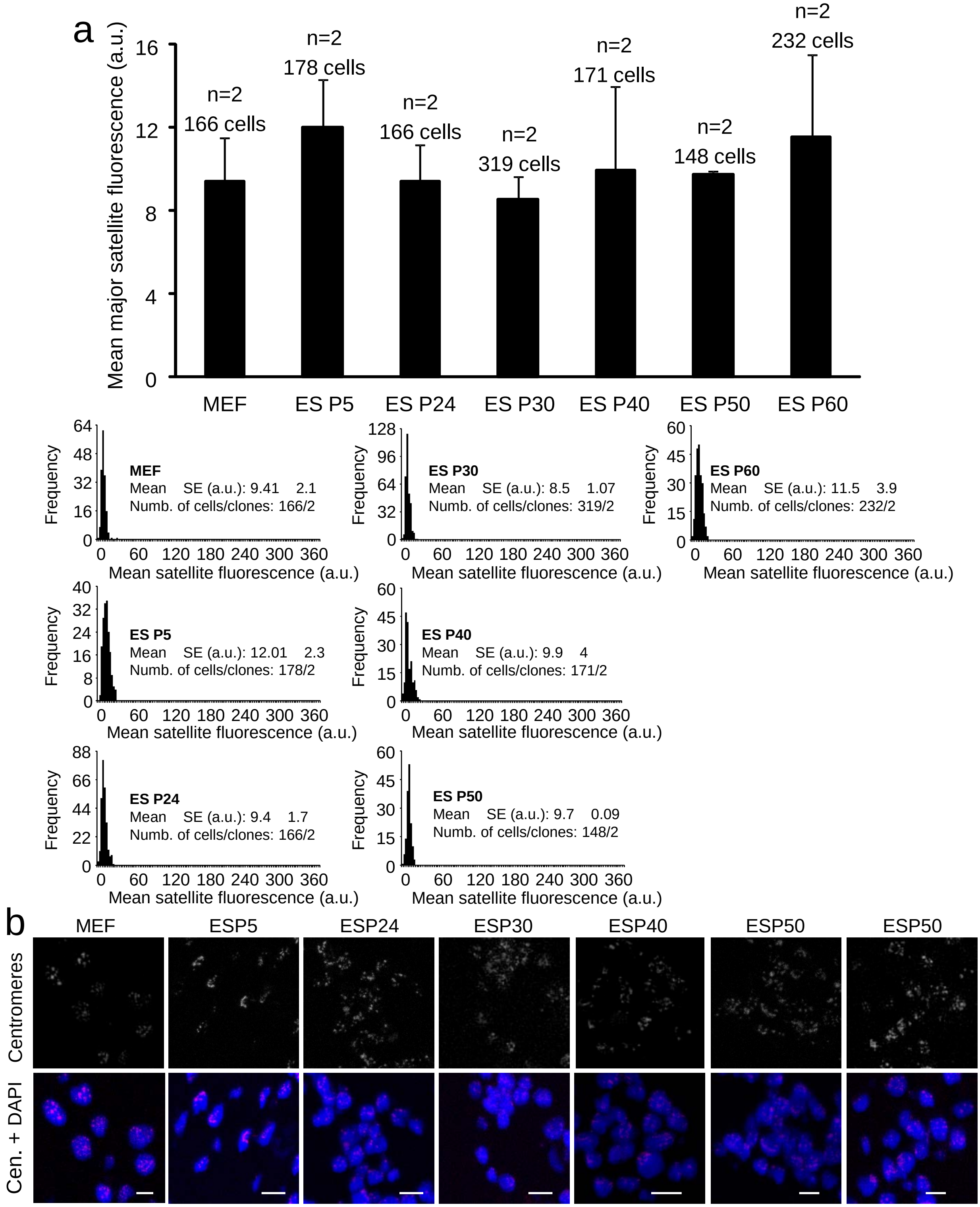

Supplementary Figure 2. Major satellite Q-FISH. a) Mean major satellite intensity for primary MEFs (passage 2) and ES cells at the indicated passages. Underneath, the histograms corresponding to the samples described above. b) Representative images of centromeric Q-FISH on the top panels, below, the merge with DAPI stain of the samples described in a. The differences in intensities were not statistically significant. Scale bar: 10µm. Student T-test with Bonferroni corrections was used to calculate the p-values.

Varela et al., Supplementary Figure 2

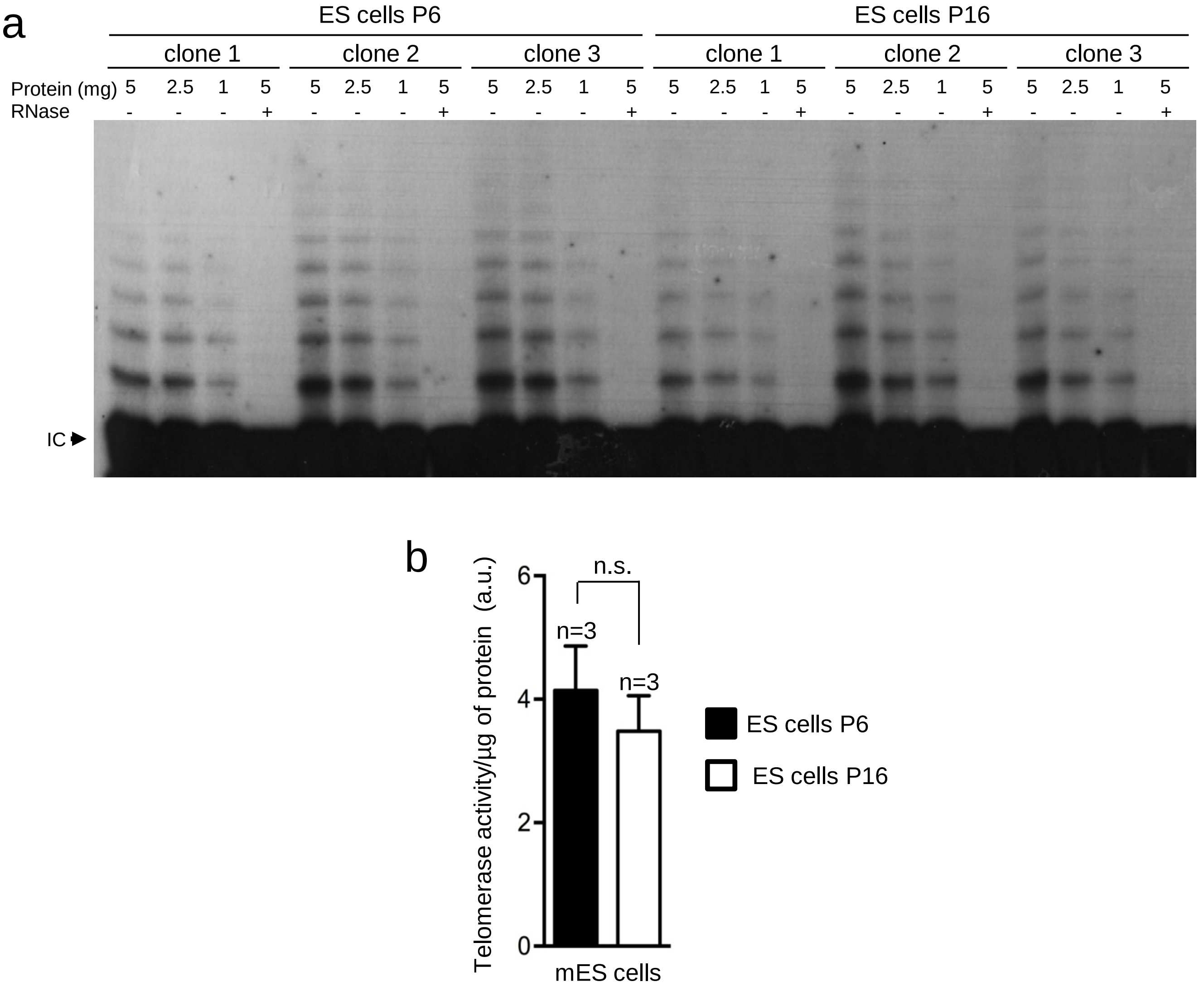

Supplementary Figure 3. Telomerase activity in early and late passage ES cells. a) The micrograph shows a telomerase activity assay form extracts of three independent clones of mES cells at passage 6 and passage 16. b) Quantification of the telomerase activity TRAP assay shown in a, in mES cells at passage 6 and passage 16.

Varela et al., Supplementary Figure 3

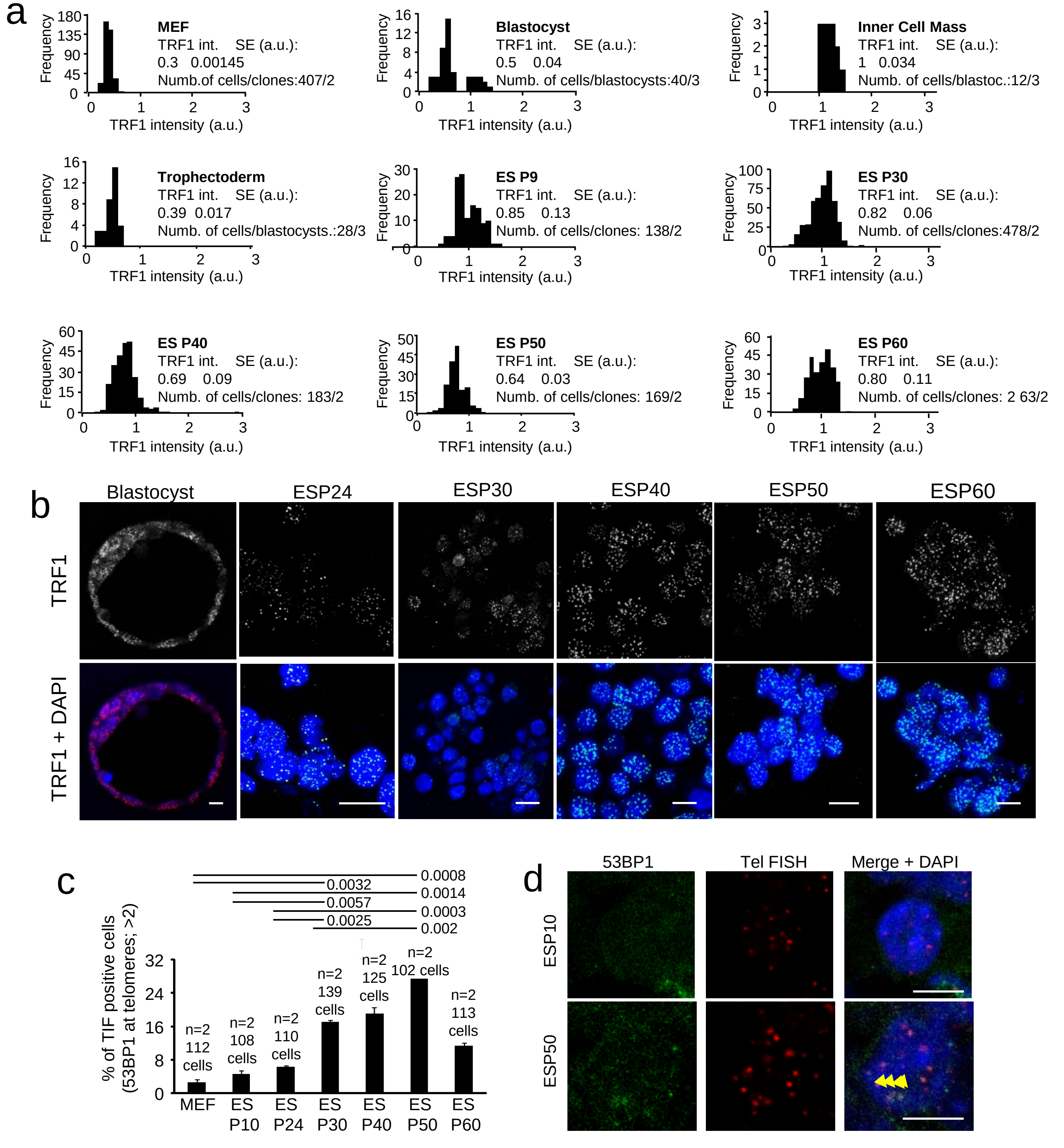

Supplementary Figure 4. TRF1 levels and DNA damage in increasing passages of ES cells. a) Histograms correspond to TRF1 intensities from blastocysts, MEF (passage 2) and ESCs at the indicated passages embedded in paraffin and analyzed by telomapping. b) Representative images of IF with anti TRF1 antibody (white) in blastocysts, or MEF (passage 2) and ES cells at different passages analyzed by telomapping. Scale bar:10 µm. c) The graph shows the percentage of cells positive for TIFs (telomere induced foci) measured by combined telomere FISH and IF using anti 53BP1 antibody in MEFs (passage 2) and ES cells at the indicated passages. d) Micrographs show combined telomere FISH with anti 53BP1 antibody in ES cells at passage 10 and 50. Scale bar: 5µm. The arrowheads indicate colocalization of 53BP1 stain with telomeres. n = the number of blastocysts or independent MEF cultures or clones of ESCs analyzed. Student T-test with the Bonferroni correction was used to calculate the p-values.

Varela et al., Supplementary Figure 4

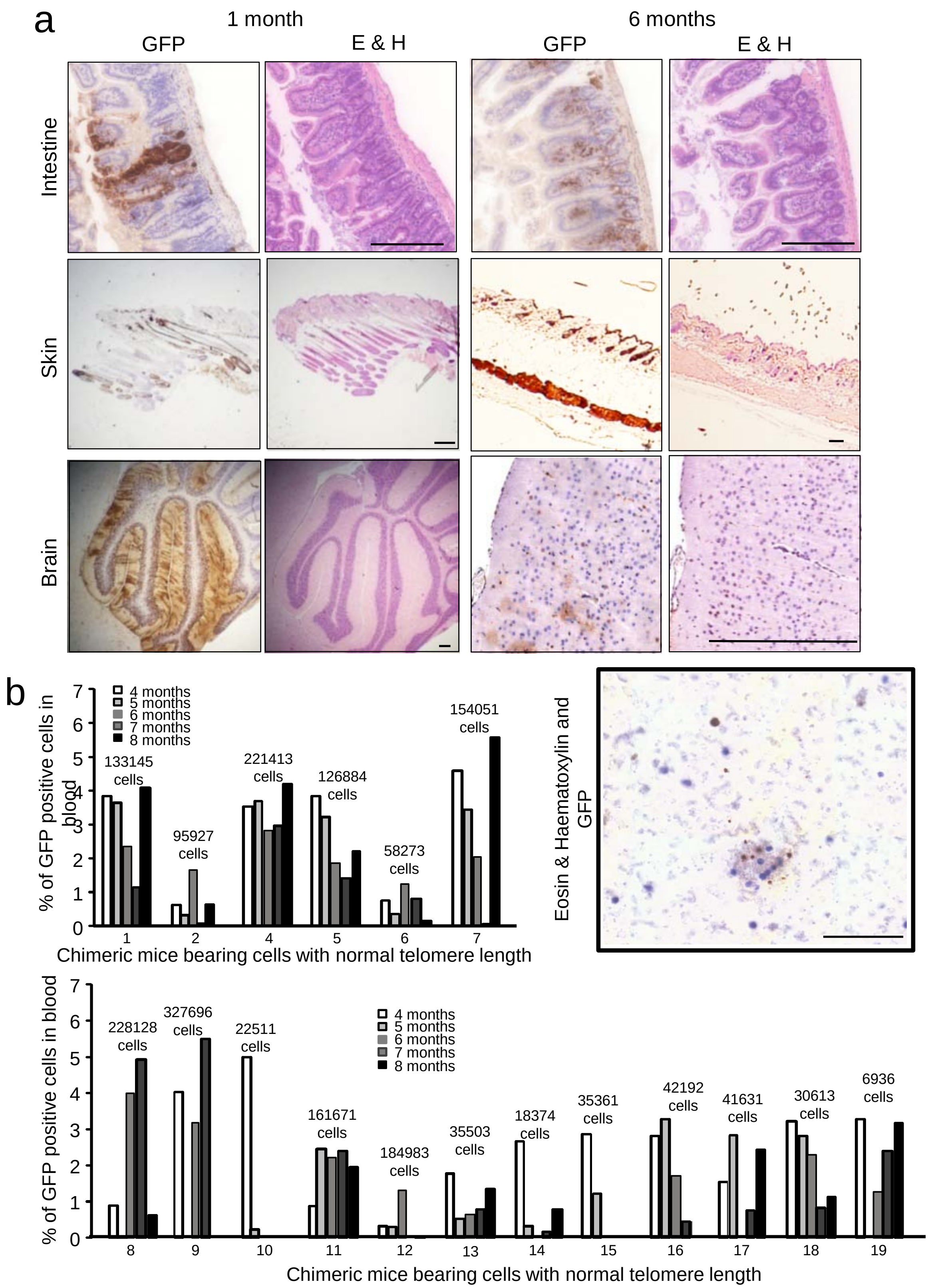

Supplementary Figure 5. Cells derived from ES cells with hyper-long telomeres contribute to mouse tissues and are maintained with aging. a) Representative images of tissues from chimeric mice bearing cells with hyper-long and normal telomeres at the indicated time points. Consecutive sections were subjected to immunohistochemistry using anti GFP antibody and stained with Eosin and Haematoxylin. Scale bar: 250 µm. b) The graph shows the percentage of GFP positive cells in blood from chimeras bearing cells with normal telomere length. Blood was extracted once per month, starting at the age of four months. A representative image of blood stained with anti GFP antibody and Eosin & Haematoxylin is shown. Scale bar: 50µm.

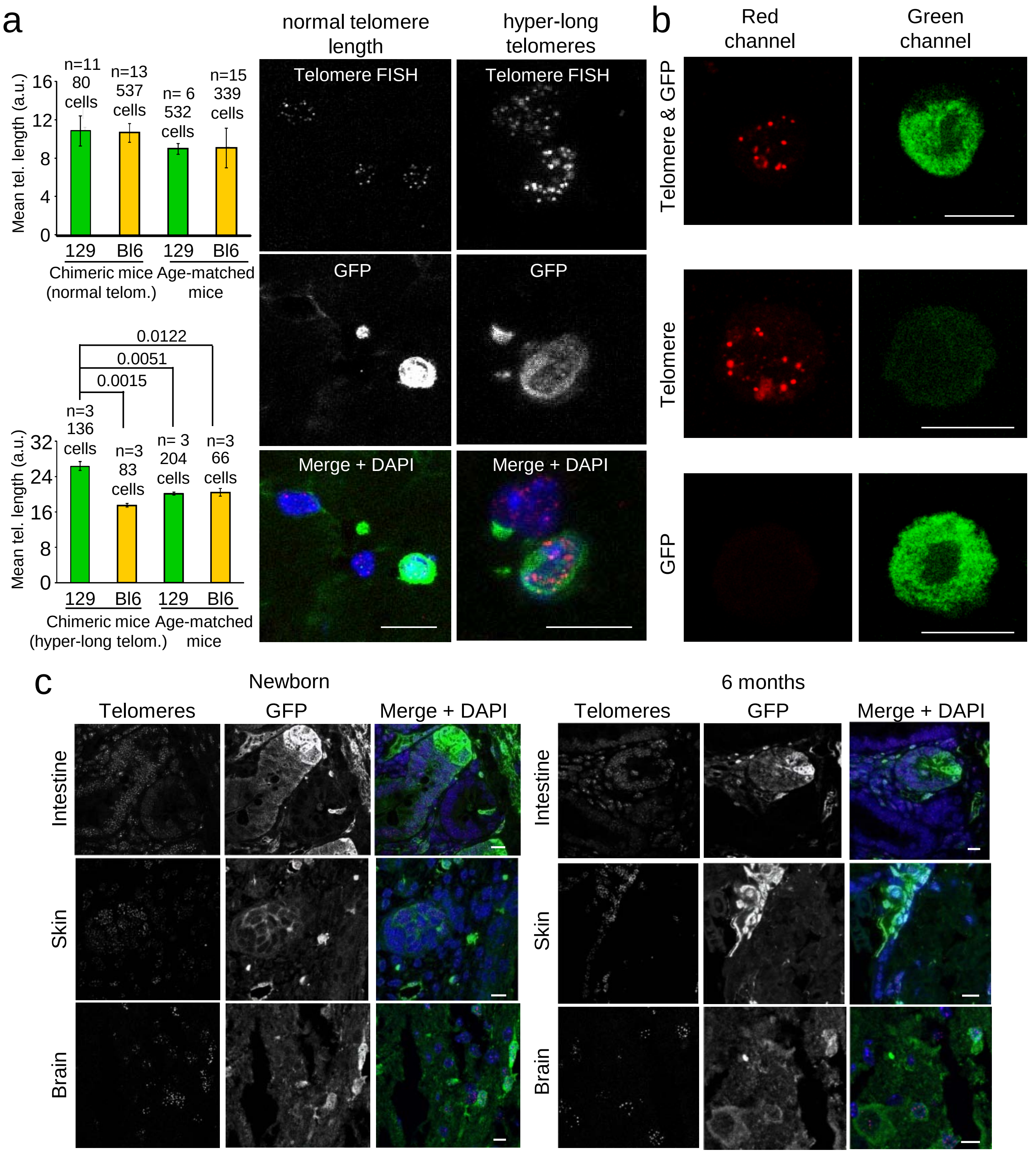

Supplementary Figure 6. Telomere length analysis in chimeric mice derived from ES cells with hyper-long telomeres. a) The graphs show mean telomere length in blood samples from chimeras bearing cells with normal (upper graph) or hyper-long telomeres (lower graph) and controls. The micrographs show telomere FISH and GFP stains and the merge with DAPI. Scale bar: 10µm. b) Micrographs show the GFP and Cy3 stains after IF with anti GFP antibody combined with FISH using a telomere probe labelled with CY3. Scale bar: 10µm. c) Representative images of telomere length from intestine, skin and brain from chimeric mice at the indicated time points. FISH combined with IF using anti GFP antibody was performed. Telomeres were analyzed by telomapping. Scale bar: 10µm. Student T-test with the Bonferroni correction was used to calculate the p-values.

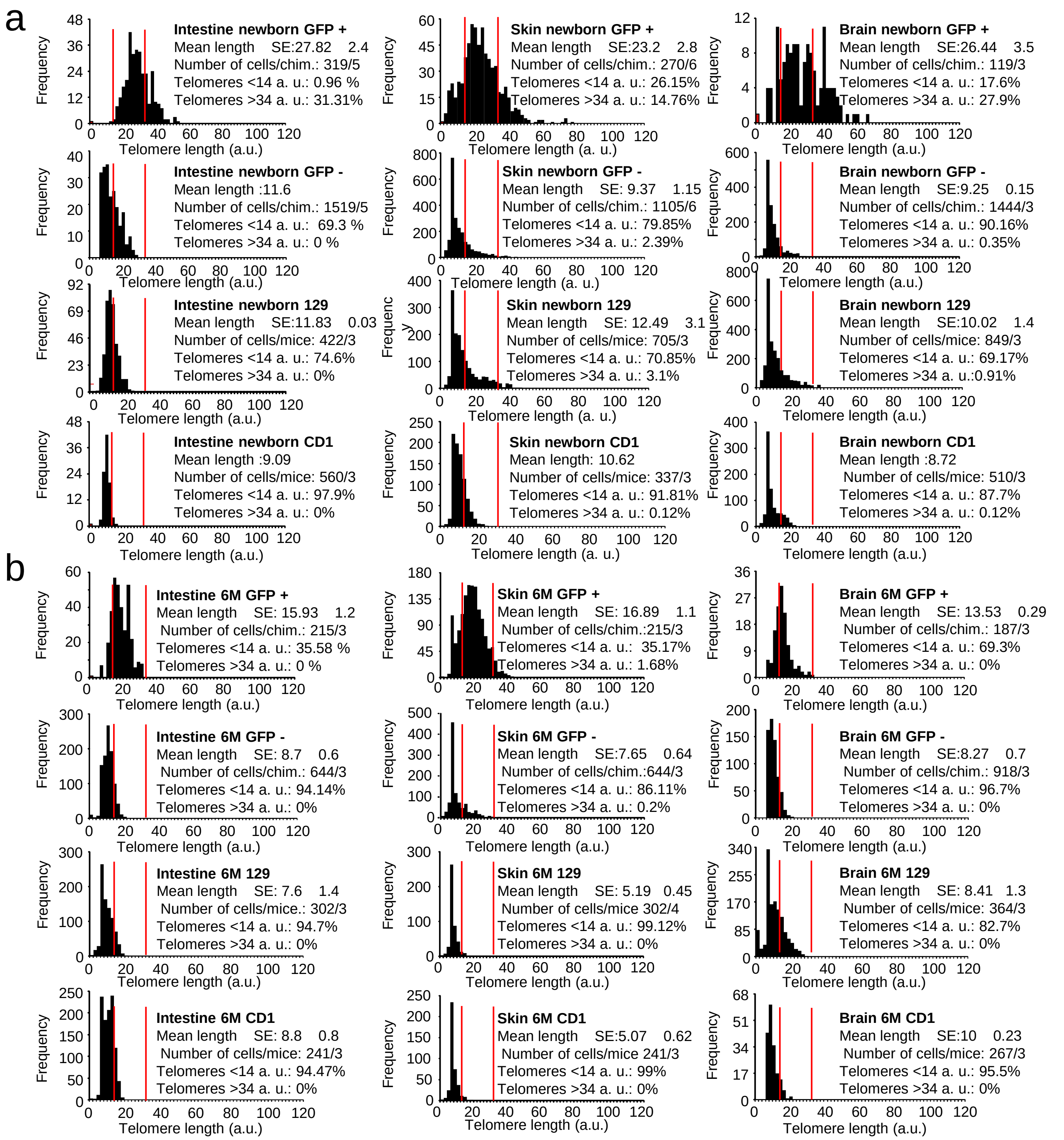

Supplementary Figure 7. Telomere length distribution in chimeric mice derived from ES cells with hyper-long telomeres and controls. a) Histograms of telomere intensity in intestine, skin and brain of newborn mice. b) Same as in a, but in six-months old mice. n= number of independent chimeric mice.

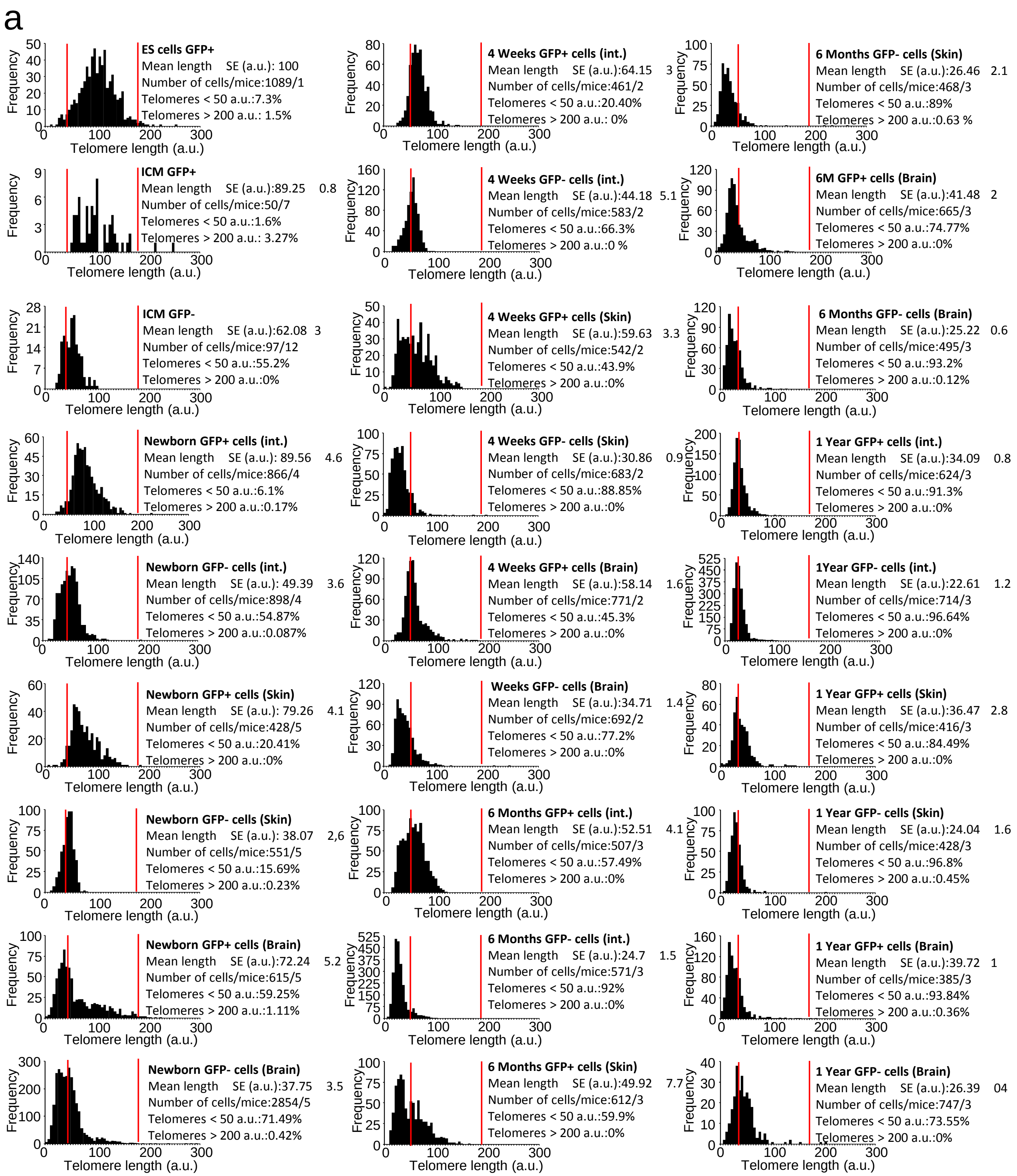

Supplementary Figure 8. Telomere length dynamics with aging in chimeric mice derived from ES cells with hyper-long telomeres. a) Histograms of telomere intensity in ES cells before injection into morulae, the inner cell mass of the blastocyst, intestine, skin and brain at the indicated time points.

a

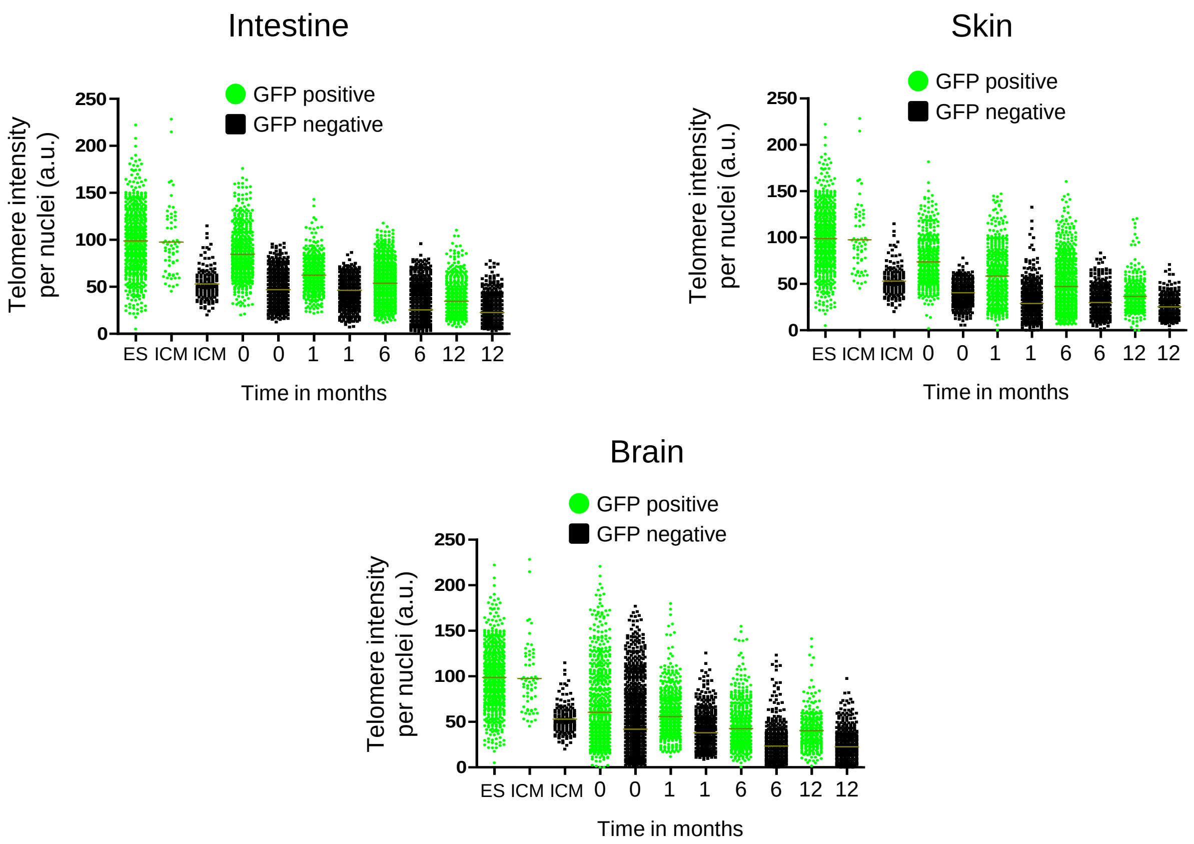

Supplementary Figure 9. Telomere length dynamics with aging in chimeric mice derived from ES cells with hyper-long telomeres. a) Scattered plots of telomere intensity in ES cells before injection into morulae, the inner cell mass of the blastocyst, intestine, skin and brain at the indicated time points. Representative graphs corresponding to two independent experiments.

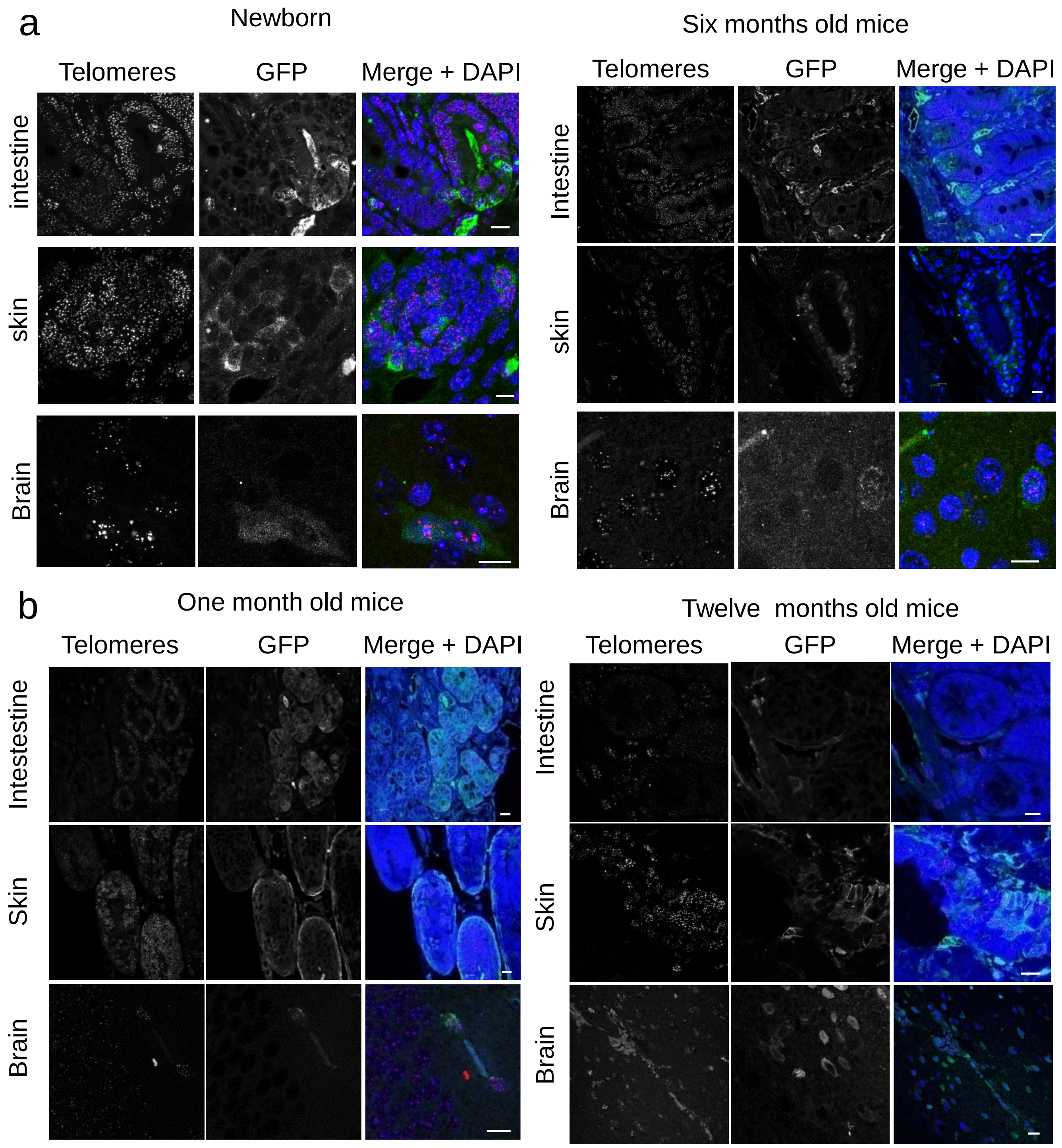

Supplementary Figure 10. Telomere length dynamics with aging in chimeric mice derived from ES cells with hyper-long telomeres. a) Representative images show telomeres by FISH combined with IF using anti GFP antibody in intestine, skin and brain in newborns and six-old mice and one- and twelve-months old mice are shown in b. Cells positive for GFP stain contained hyper-long telomeres. Bar=10µm.

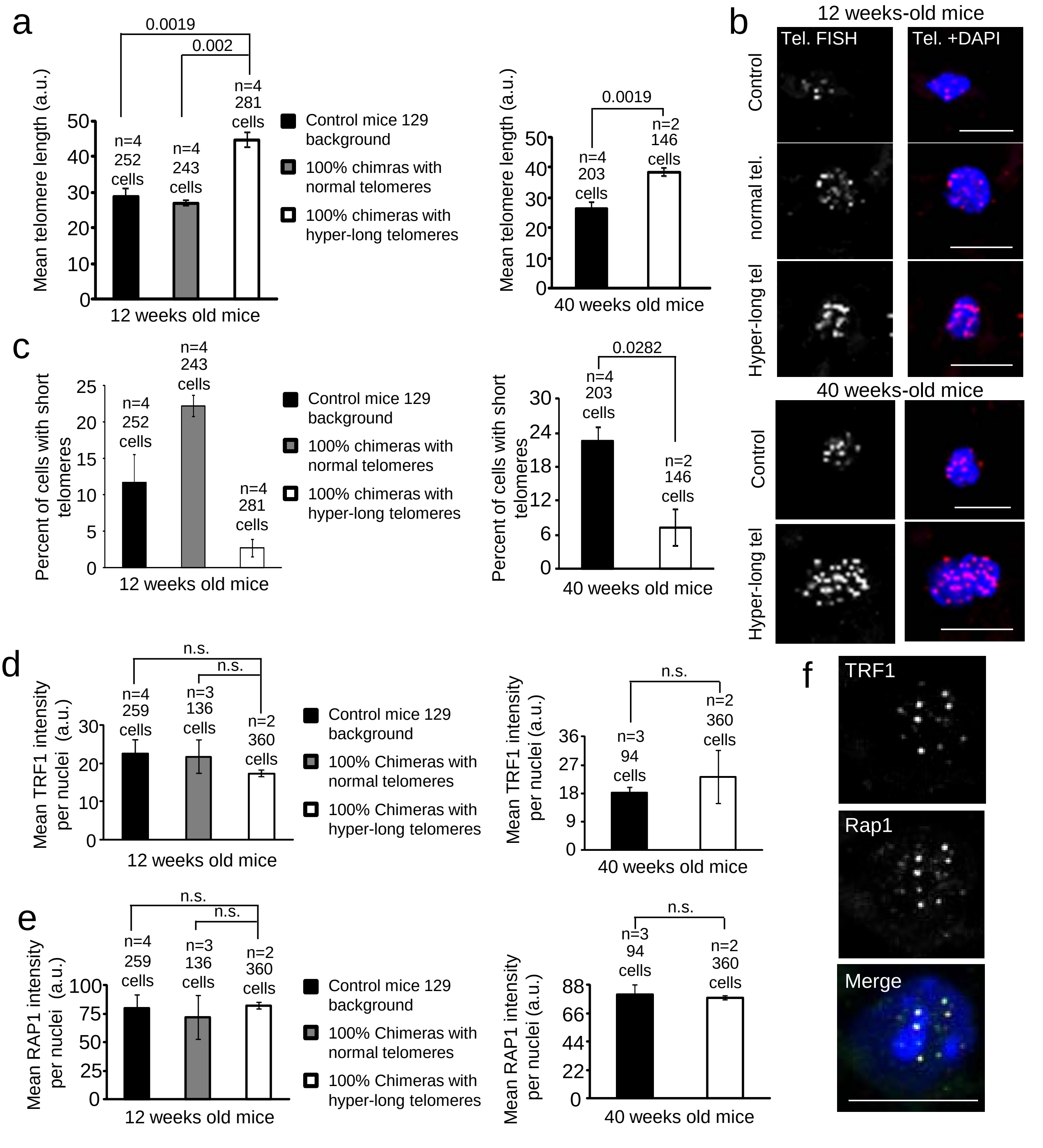

Supplementary Figure 11. Telomere length and shelterin dynamics with aging in 100% chimeras derived from ES cells with hyper-long telomeres. a)The graphs show mean telomere length in blood samples from chimeric mice of 100% of chimerism and age-matched controls at the indicated time points. b) Representative images of blood samples as described in a at the indicated time points subjected to quantitative FISH. Scale bar: 10µm. c) The graphs show the percent of cells with short telomeres in the blood samples described in a at the indicated time points. Note that in chimeric mice with hyper-long telomeres the accumulation of short telomeres is lower. d) The graphs represent the mean TRF1 intensity in blood samples described in a. The samples were subjected to immunofluorescence with anti TFR1 and anti RAP1 antibodies. e) The graphs show mean RAP1 intensity in the blood samples described in a subjected to immunofluorescence as described in d. f) Representative images of TRF1 and RAP1 stain in the samples described in a. Scale bar: 10µm. Student T-test with the Bonferroni correction was used to calculate the p-values.

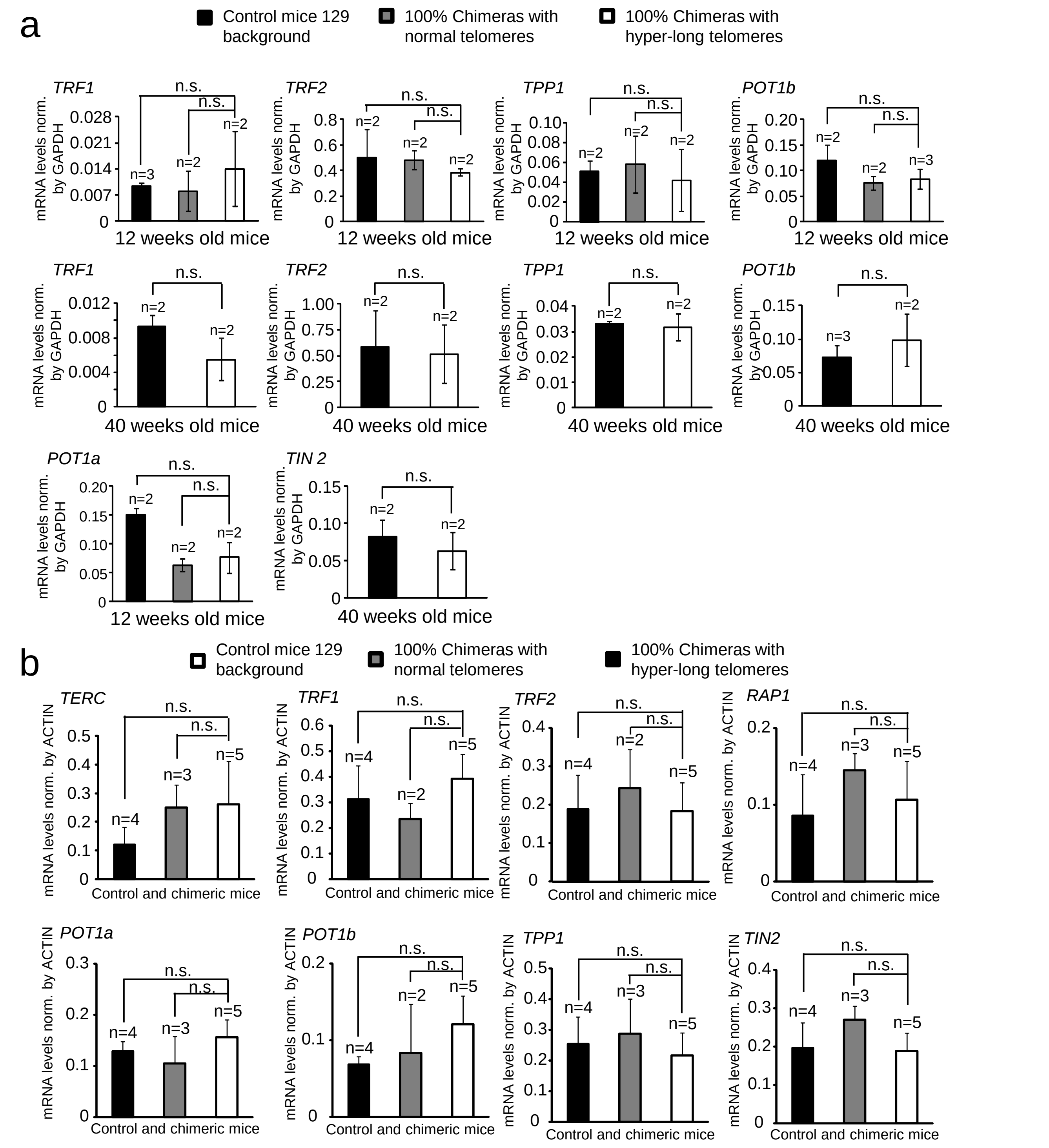

Supplementary Figure 12. Telomerase and shelterin mRNA levels in blood and skin in 100% chimeras derived from ES cells with hyper-long telomeres. a) The graphs show the mRNA levels of shelterins in blood samples from chimeric mice of 100% of chimerism and age-matched controls at the indicated time points. b) the graphs show the mRNA levels of shelterins and telomerase in skin from chimeric mice with normal or hyper-long telomeres and age-matched controls. Student T-test with the Bonferroni correction was used to calculate the p-values.

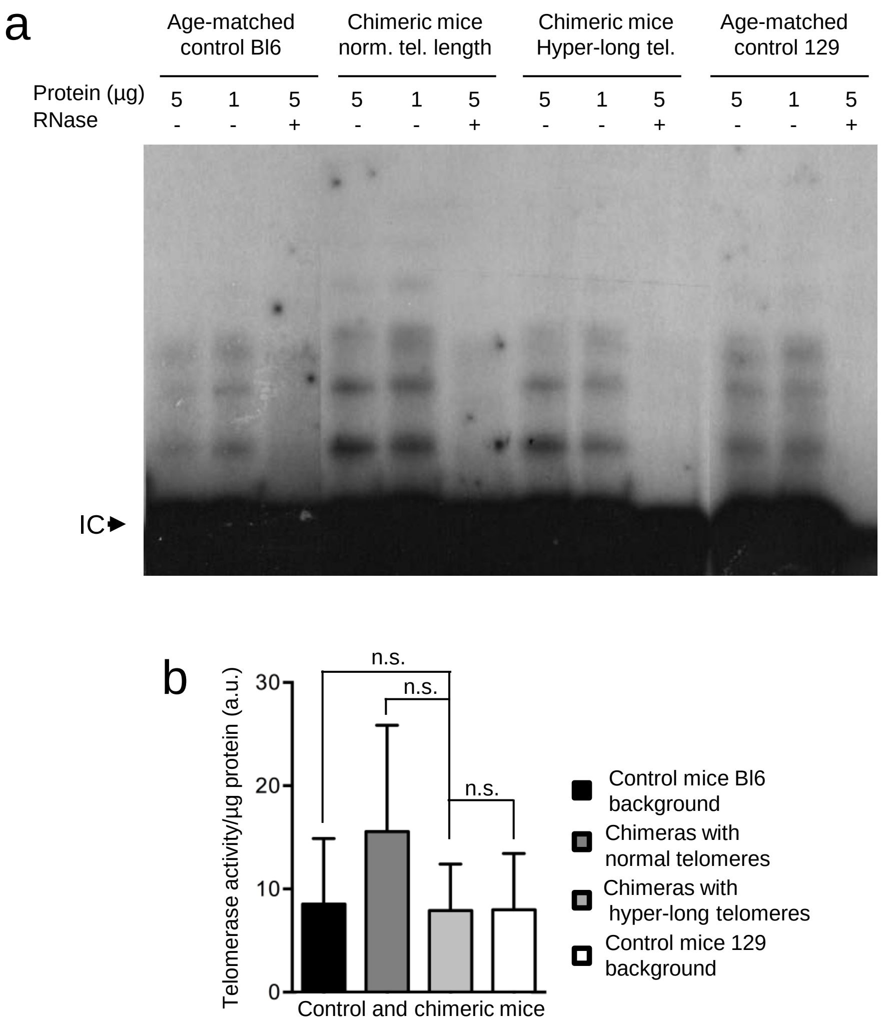

Supplementary Figure 13. Telomerase activity in spleen from chimeras derived from ES cells with hyper-long telomeres. a) Analysis of telomerase activity in spleen of chimeric mice with normal or hyper-long telomeres and age-matched controls. b) Quantification of the telomerase activity TRAP assay shown in a, in spleen from chimeric mice with normal or hyper-long telomeres and controls. n= number of independent chimeric mice. Student T-test with the Bonferroni correction was used to calculate the p-values.

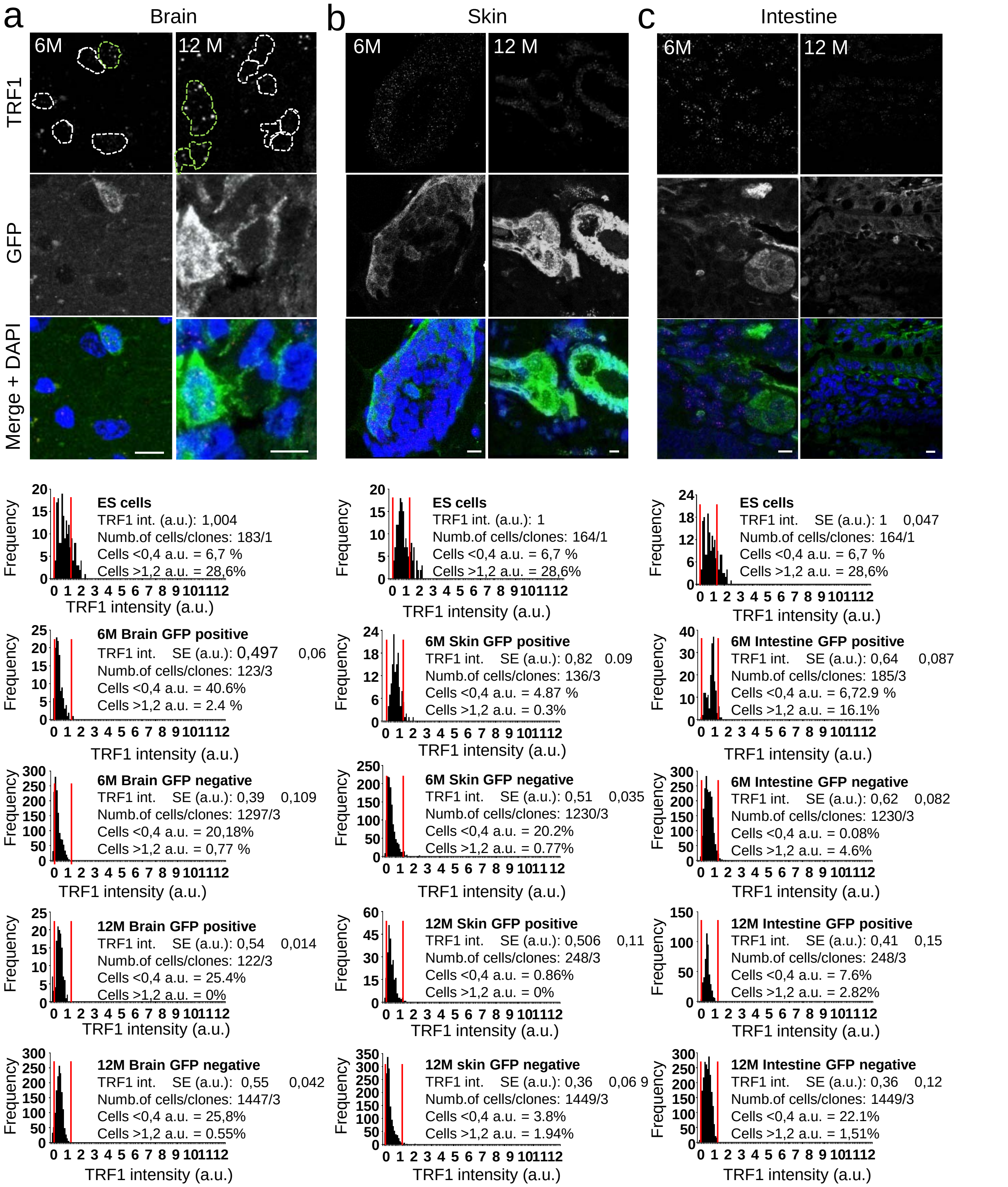

Supplementary Figure 14. Analysis of TRF1 and γH2AX intensity in tissues from chimeras derived from ES cells with hyper-long telomeres. a) Representative images show TRF1 and GFP stains and the merge with DAPI in brain tissue. Underneath, the histograms corresponding to ES cells with hyper-long telomeres that were used for microinjection to generate chimeras, and 6 or 12 months tissues from brain. Scale bar:10µm. Similar images and histograms are shown for Skin in b and for intestine in c. Scale bar:10µm. The cells were depicted in the image (dotted line).

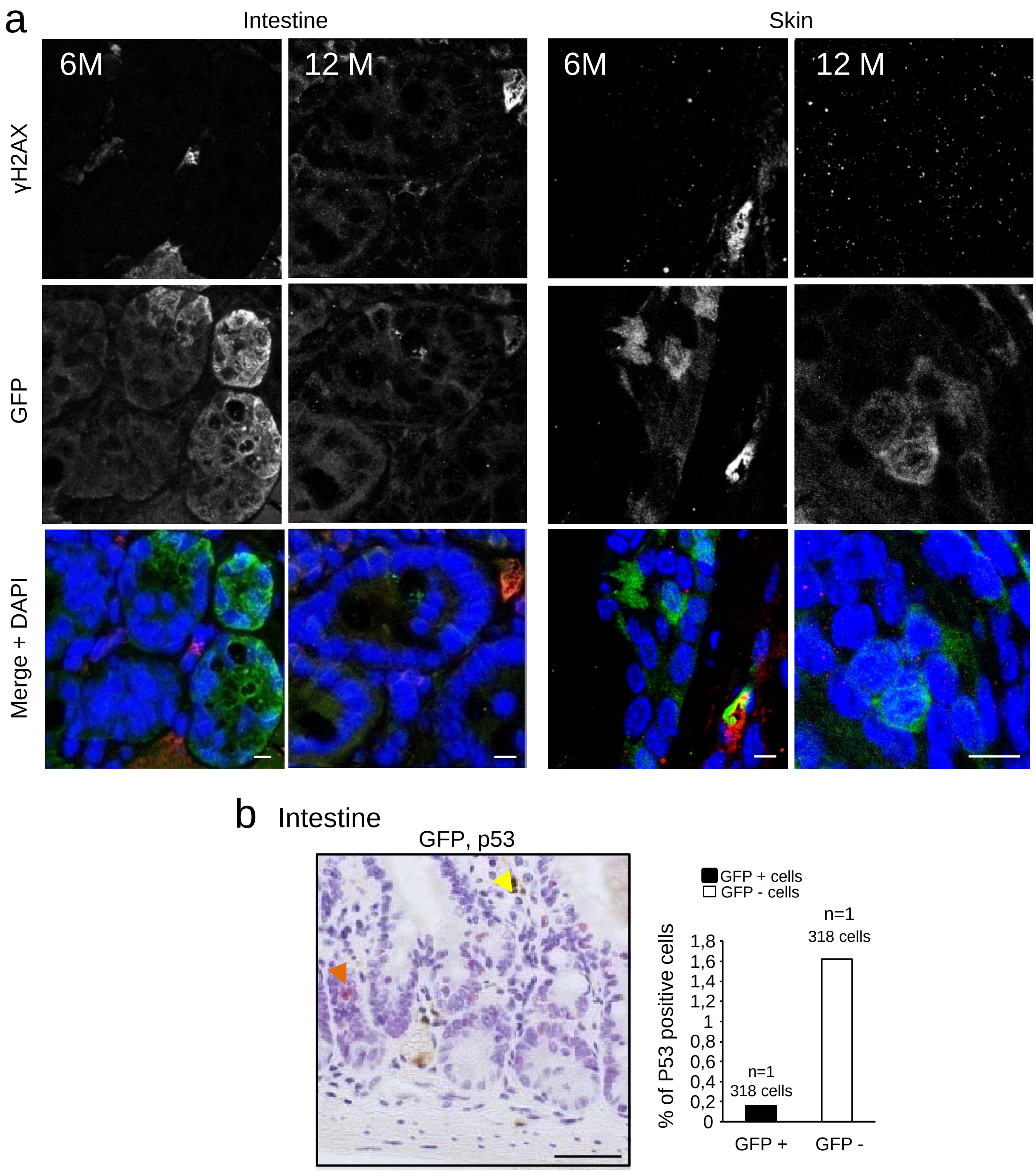

Supplementary Figure 15. Analysis γH2AX intensity and p53 levels in tissues from chimeras derived from ES cells with hyper-long telomeres. a) Representative images show γH2AX and GFP stains, and the merge with DAPI in skin and intestinal tissues at the indicated time points. Scale bar: 10µm. b) The micrograph shows intestine from chimeric mice bearing cells with hyper-long (GFP positive) and normal telomeres (GFP negative). Sections were subjected to immunohistochemistry using anti GFP and P53 antibodies and stained with Eosin and Haematoxylin. The orange arrowhead indicates the p53 positive cells and the yellow arrowhead indicates the GFP positive cells. The graph shows the percentage of P53 positive cells in the GFP positive and negative cells. Scale bar: 50 µm.

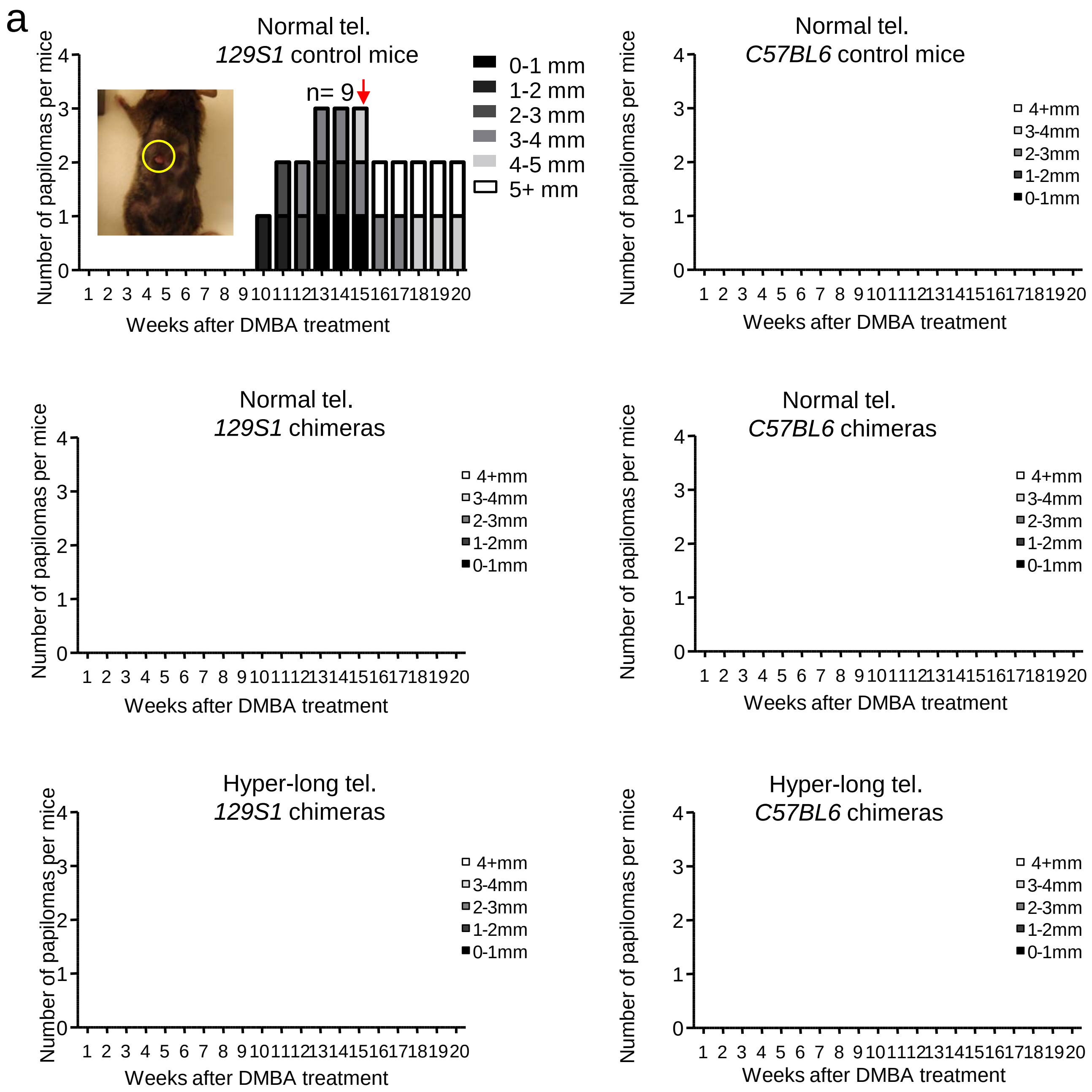

Supplementary Figure 16. Skin chemical carcinogenesis in chimeras derived from ES cells with normal or hyper-long telomeres and controls. a) The graphs show the size of skin papillomas after treatment with a carcinogenic agent (DMBA-TPA) for 15 weeks, chimeric mice bearing hyper-long telomeres (note that areas of skin of the *129S1* and *C57Bl6* backgrounds have been plotted separately), chimeric mice with normal telomere length (note that areas of skin of the *129S1* and *C57Bl6* backgrounds have been plotted separately) and age-matched controls. The red arrow indicates the end of TPA treatment.

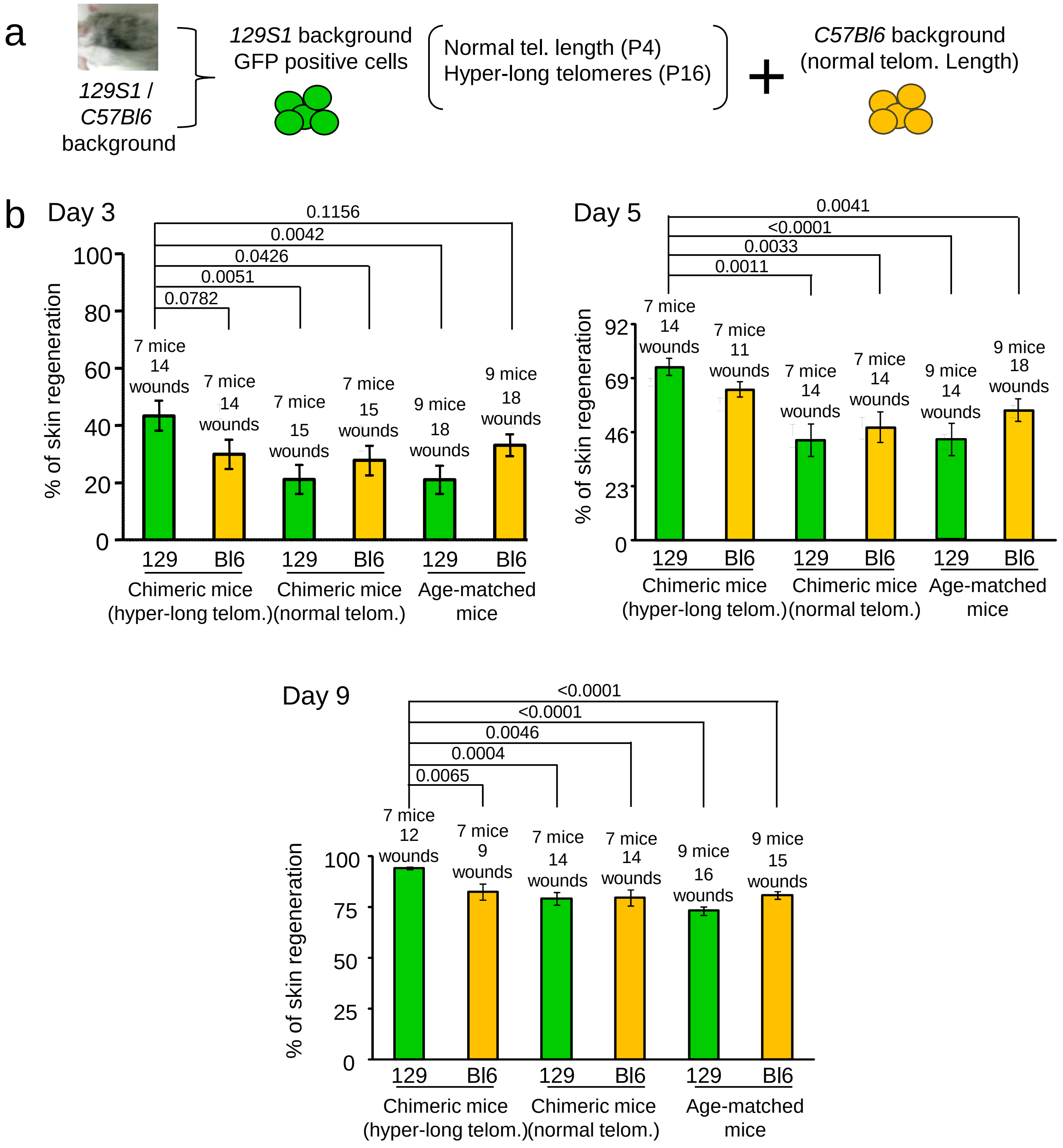

Supplementary Figure 17. Analysis of skin wound healing in chimeras derived from ES cells with normal or hyper-long telomeres and controls. a) The scheme shows the background and telomere lengths of the cells composing the chimeras used for this experiment. b) The graphs show the percent of skin regenerated after performing 4mm wounds in chimeric mice bearing normal telomere length or hyper-long telomeres as well as in age-matched mice of the same genetic backgrounds.

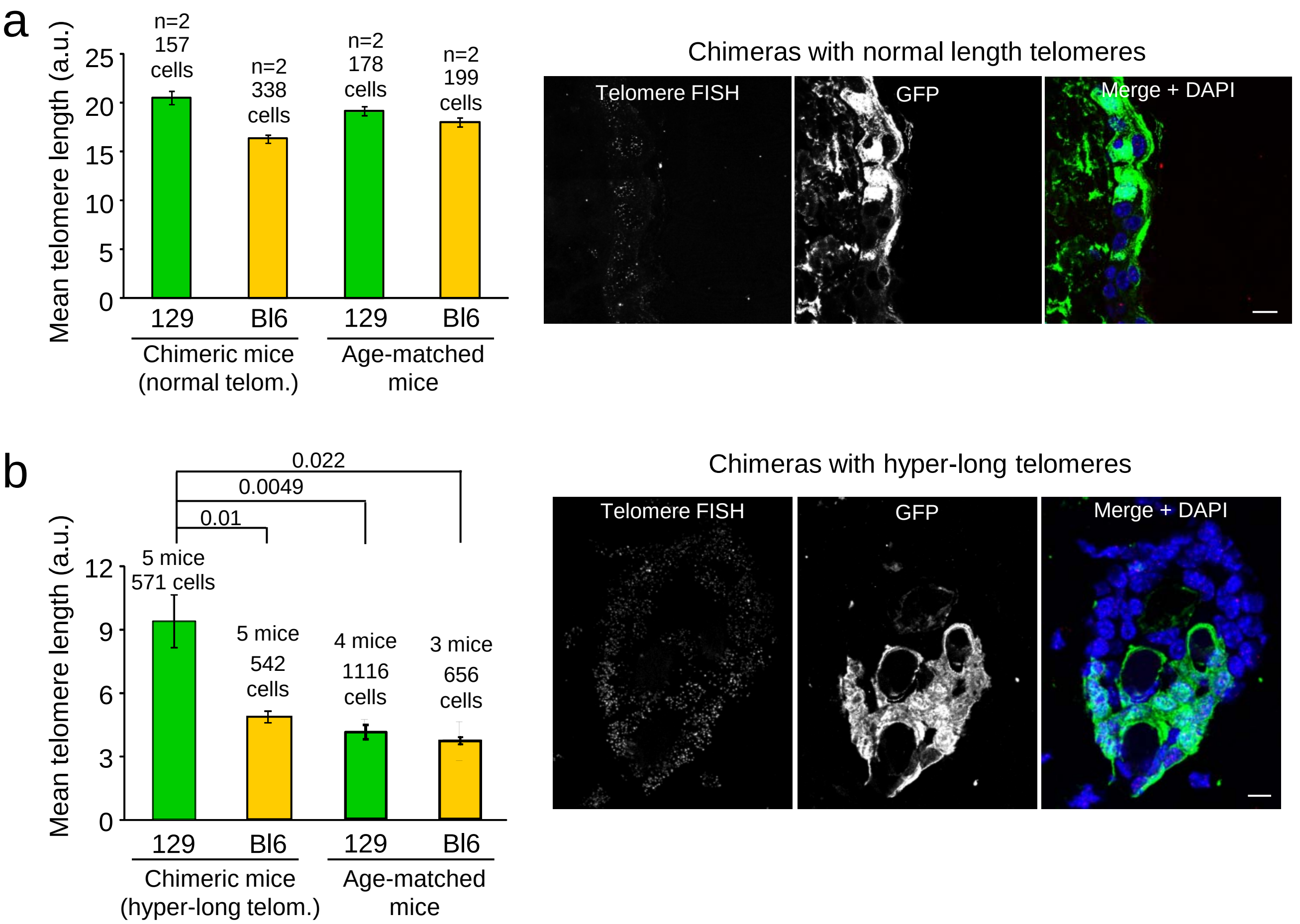

Supplementary Figure 18. Analysis of telomere length in skin extracted during the wound healing procedure in chimeras derived from ES cells with normal or hyper-long telomeres. a) The graph shows mean telomere length in skin from chimeric mice and age-matched controls. The micrographs show telomere FISH and GFP stains, and the merge with DAPI. Scale bar: 10µm. b) The graph shows mean telomere length in the skin of chimeras bearing hyper-long telomeres and age-matched controls. The micrographs show telomere FISH and GFP stains, and the merge plus DAPI. Bar=10µm.

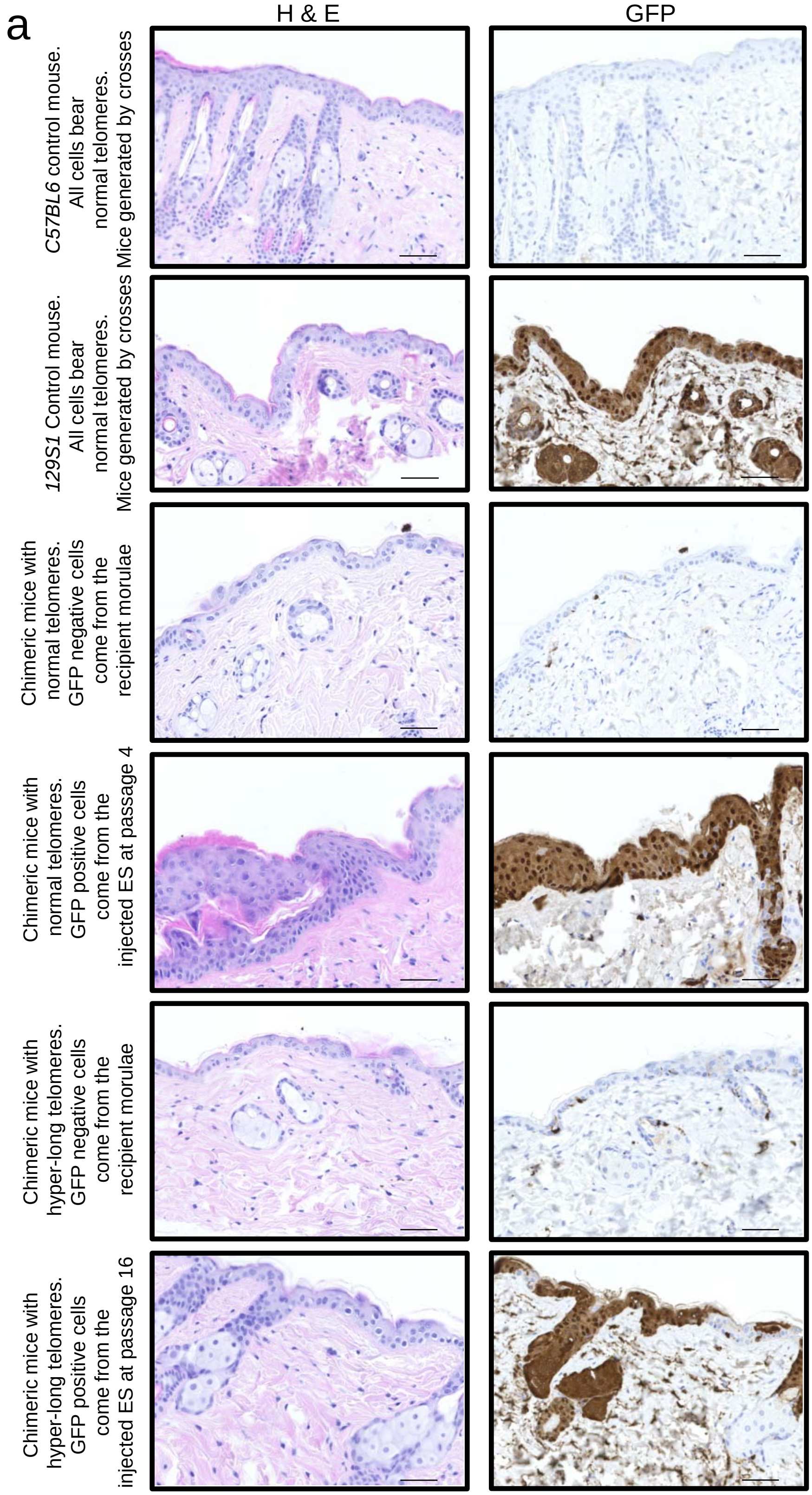

Supplementary Figure 19. Analysis of skin wound healing in chimeras derived from ES cells with normal or hyper-long telomeres. e) Eosin and Haematoxylin as well as GFP stains on sections of skin extracted from mice when the wound had healed. Bar=50µm.

Supplementary Table 1. RNA sequencing of ES cells at passage 16 versus passage 6

Gene name	Log2 fold change	P value	Function
Sox18	4.455160405	1.73E-224	Embryonic and postnatal angiogenesis (1)
Sox17	-1.185658886	5.18913E-12	Embryonic and postnatal angiogenesis (2)
Zbtb48	-0.969303187	4.3873E-08	POK family protein (3)
Chst15	-0.8167311010.16707432	1.01643E-06	Sulfotransferase (4)
Jph4	-0.6540687230.13873356	2.42225E-06	Junctional membrane complexes (5)

In red genes that were upregulated at passage 16 compared to passage 6 and in green genes that were downregulated at passage 16 compared to passage 6.

Supplementary Table 2. List of primers used for qPCR analysis

Gene name	Sequence
RAP1-F	5´-AAGGACCGCTACCTCAAGCA-3`
RAP1-R	5´-TGTTGTCTGCCTCTCCATTC-3´
TRF1-F	5´-CTAAGGATAGGCCAGATGCCA-3´
TRF1-R	5´-CTGAAATCTGATGGAGCACGT-3´
TRF2-F	5´-AGAGCCAGTGGAAAAACCAC-3´
TRF2-R	5´-ATGATGGGGATGCCAGATTA-3´
TIN2-F	5´-TCGGTTGCTTTGCACCAGTAT-3´
TIN2-R	5´-GCTTAGCTTTAGGCAGAGGAC-3´
POT1a-F	5´-AAACTATGAAGCCCTCCCCA-3´
POT1a-R	5´-CGAAGCCAGAGCAGTTGATT-3´
POT1b-F	5´-TTGGAAGCCATTCAGACCTC-3´
POT1b-R	5´-GGTTGACATCTTTCCACCTCAT-3´
TPP1-F	5´-ACTTGTGTCAGACGGAACCC-3´
TPP1-R	5´-CAACCAGTCACCTGTATCC-3´
TERC-F	5´-TCATTAGGTGTGGGTTCTGGT-3´
TERC-R	5´- TGGAGCTCCTGCGCTGACGTT-3´

Supplementary References

(1) François, M. et al. Sox18 induces development of the lymphatic vasculature in mice. Nature 456, 643-648 (2008).

(2) Zhou, Y. et al. Sox7, Sox17 and Sox18 cooperatively regulate vascular development in the mouse retina. PLoS One 10, DOI:10.1371/journal.pone.0143650 (2015).

(3) Yoon, J.H. et al. Human Krüppel-related 3 (HKR3) is a novel transcription activator of alternate reading frame. The Journal of Biological Chemyistry 289, 4018-4031.

(4) Wu, Z.L. et al. Detection of specific glycosaminoglycans and glycan epitopes by in vitro sulfation using recombinant sulfotransferases. Glycobiology 21, 625-633 (2011).

(5) Moriguchi, S. et al. Finctional uncoupling between Ca2+ release and afterhyperpolarization in mutant hippocampal neurons lacking junctophilins. Proc Natl Acad Sci USA 103, 10811-10816.
